# A multi-calibrated mitochondrial phylogeny of extant Bovidae (Artiodactyla, Ruminantia) and the importance of the fossil record to systematics

**DOI:** 10.1186/1471-2148-13-166

**Published:** 2013-08-08

**Authors:** Faysal Bibi

**Affiliations:** 1 Museum für Naturkunde, Leibniz Institute for Research on Evolution and Biodiversity at the Humboldt University Berlin, Invalidenstrasse 43, Berlin 10115, Germany; 2 Current address: Department of Mammalogy, American Museum of Natural History, 200 Central Park West, New York, NY 10024, USA

**Keywords:** Fossil calibration, Ruminantia, Bayesian analysis, Phylogenetic systematics, Neogene

## Abstract

**Background:**

Molecular phylogenetics has provided unprecedented resolution in the ruminant evolutionary tree. However, molecular age estimates using only one or a few (often misapplied) fossil calibration points have produced a diversity of conflicting ages for important evolutionary events within this clade. I here identify 16 fossil calibration points of relevance to the phylogeny of Bovidae and Ruminantia and use these, individually and together, to construct a dated molecular phylogeny through a reanalysis of the full mitochondrial genome of over 100 ruminant species.

**Results:**

The new multi-calibrated tree provides ages that are younger overall than found in previous studies. Among these are young ages for the origin of crown Ruminantia (39.3–28.8 Ma), and crown Bovidae (17.3–15.1 Ma). These are argued to be reasonable hypotheses given that many basal fossils assigned to these taxa may in fact lie on the stem groups leading to the crown clades, thus inflating previous age estimates. Areas of conflict between molecular and fossil dates do persist, however, especially with regard to the base of the rapid Pecoran radiation and the sister relationship of Moschidae to Bovidae. Results of the single-calibrated analyses also show that a very wide range of molecular age estimates are obtainable using different calibration points, and that the choice of calibration point can influence the topology of the resulting tree. Compared to the single-calibrated trees, the multi-calibrated tree exhibits smaller variance in estimated ages and better reflects the fossil record.

**Conclusions:**

The use of a large number of vetted fossil calibration points with soft bounds is promoted as a better approach than using just one or a few calibrations, or relying on internal-congruency metrics to discard good fossil data. This study also highlights the importance of considering morphological and ecological characteristics of clades when delimiting higher taxa. I also illustrate how phylogeographic and paleoenvironmental hypotheses inferred from a tree containing only extant taxa can be problematic without consideration of the fossil record. Incorporating the fossil record of Ruminantia is a necessary step for future analyses aiming to reconstruct the evolutionary history of this clade.

## Background

Phylogenetic studies based on genomic data provide an unparalleled tool for the reconstruction of phylogenetic relationships among organisms. However, considering that the overwhelming majority of species that have ever existed on Earth are now extinct, attempts to reconstruct evolutionary history must necessarily consider the fossil record. A key challenge of contemporary phylogenetic methods, therefore, is to combine data from the fossil record with genomic data from living organisms [1]. One of the main contributions of the fossil record to molecular phylogenetics has been the provision of fossil calibrations for molecular rates of evolution that in turn allow for estimates of the timing and rate of cladogenetic events. Molecular age estimation is of particular importance for evolutionary events for which the fossil record is incomplete, ambiguous, or even totally unknown. However, the paleontological data used for molecular rate calibration are often misunderstood and misapplied, resulting in poor (and wildly varying) age estimates [1-7]. The problem stems in part from the opacity of paleontological literature to non-specialists, as well as to the fact that fossils are not always properly assigned to either the stem group or the crown clade of a taxon [7].

One clade that has been the subject of numerous studies of molecular age estimation is Bovidae, or the clade comprising antelopes, oxen, sheep, and goats. With over 130 living species, Bovidae is the most diverse clade of large mammals alive today, and includes almost half of all living species included in Artiodactyla (= Cetartiodactyla). Bovids are ecologically and geographically diverse, spanning the Rocky Mountains to the African rainforest, and their fossil record is abundant and spans the last 18 million years. Interest in bovid phylogenomics is widespread, covering subjects as diverse as Neogene phylogeography to the reconstruction of human domestication practices in the late Holocene. Age-calibrated trees of Bovidae are also relevant to studies seeking to establish rates of evolution within a larger context of ruminant and mammalian evolution, including, for example, the terrestrial origins of whales e.g. [8,9]. Though the fossil record of Bovidae and Ruminantia is extensive, difficulties of phylogenetic resolution mean that many topological relationships and phylogeographic reconstructions have come from molecular phylogenies of extant taxa. Similarly, many of the evolutionary divergences within Ruminantia have been dated mainly through molecular age estimates [8,10,11]. However, most of these studies have relied on a single or a few (often incorrectly applied) fossil calibration points that are often far removed from many of the nodes being dated. The large number and high diversity of species within ruminants means that molecular rate variations are a real possibility, and that the use of a larger number of fossil calibration points distributed throughout the tree should result in better age estimates that in turn better reflect the evolutionary history of the clade.

In an attempt to test this idea, I here identify 16 fossil calibration points relevant to the phylogeny of Bovidae and Ruminantia, and apply these, together and individually, to a recently published matrix of the complete mitochondrial genome of over 100 ruminant species [8]. Running each of the 16 calibration points alone in independent analyses provides a sense of the degree to which calibration choice can affect molecular age estimates as well as any effects on tree topology. Running all available calibrations together anticipates possible variations in molecular rates, and helps provide new age estimates reflecting a consensus of a larger portion of the fossil record. I also use the results of these analyses to highlight current areas of conflict between genomic and morphological approaches to evolutionary reconstruction, and to promote the incorporation of increasing amounts of paleontological information into molecular phylogenetic approaches.

In what follows, references to Pleistocene refer to the recently redefined Pliocene-Pleistocene boundary at 2.588 Ma (previously at 1.8 Ma) [12]. The term crown clade refers to a node-based clade originating with the last common ancestor of two or more extant species or organisms [13]. The stem group, or stem lineage, is the ancestral lineage leading to a crown group. The branch-based clade that comprises a crown clade plus the stem leading to it is the ‘total’ or ‘pan-’ clade [13]. I use the order name Artiodactyla (rather than Cetartiodactyla) as Cetaceans are nested within Artiodactyla and not its sister group (i.e. whales are artiodactyls too) [9,14]. I follow long-standing classifications that recognize between about 130 and 145 extant bovid species [15-18], rather than a recent treatment proposing 279 species [19].

## Results

**Figure 1:**
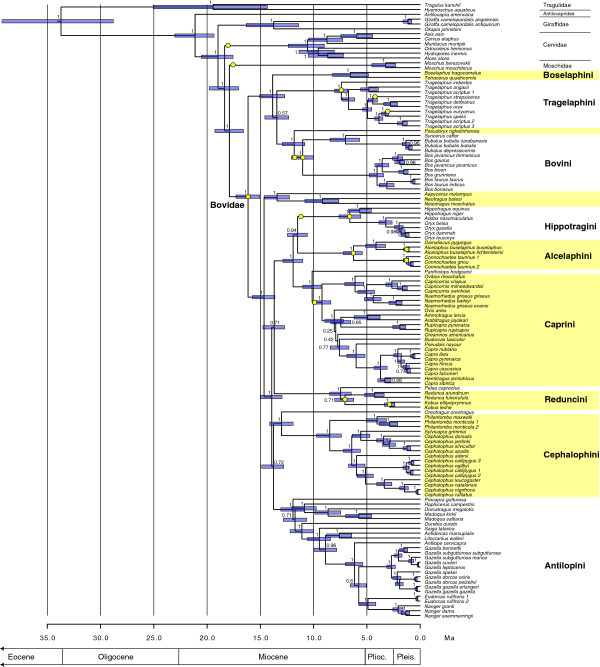
**Tree resulting from Bayesian analysis of the full mitochondrial genome of 127 ruminants using 16 fossil calibration points (yellow circles).** Calibrations along branches indicate stem calibrations. Node bars represent 95% intervals and node values are posterior probabilities. Names of terminal taxa follow those in ref. [8].

The 16 fossil calibrations used are listed in Table 1 and full details on each are given as Additional file 1. The maximum clade credibility (MCC) tree summarizing the trees found from the analysis using 16 calibration points is shown in Figure 1 (and Additional file 2: Table S1). The topology and divergence age estimates of this tree differ in a few respects from those of the recent analyses of Artiodactyla (= Cetartiodactyla) by Hassanin et al. [8], the source of the data matrix used in this study. MCC trees from the runs using each calibration point alone and the run using no calibration priors and random starting trees are available (with full metadata) online through TreeBase. Trees resulting from the different calibrated analyses show slight variations in topology (detailed below) indicating that choice of calibration node did have some effect on tree topology. The topology of the analysis using no calibrations and random starting trees is the same as those of some of the calibrated analyses, indicating that use of a starting tree did not have a significant effect on final tree topology.

**Table 1:** Fossil calibration data

**Calibrated node / branch**	**Age Ma**	**Age type**	**95% range**	**Fossil taxon**	**Fossil reference**	**Chronological reference**
Stem Moschidae	23	Minimum	23.0–28.4 Ma	*Dremotherium feignouxi*	Janis and Scott [20]; Gentry et al. [21]	Gradstein et al. [22]
Crown Bovidae	18	Approximate	16–20 Ma	*Eotragus noyei*	Solounias et al. [23]	Solounias et al. [23]
Stem Cervidae	16.6	Minimum	16.6–28.4 Ma	*Procervulus praelucidus Ligeromeryx praestans Acteocemas infans*	Gentry et al. [21]	Agustí et al. [24]
Stem Bovini	10.2	Minimum	10.2-16 Ma	*Selenoportax vexillarius*	Pilgrim [25]; Bibi [26]; Bibi [27]	Barry et al. [28]; Badgley et al. [29]
Stem Caprini	8.9	Minimum	8.9-13.0 Ma	*Aragoral mudejar*	Alcalá and Morales [30]	Van Dam et al. [31]
Crown Bovini	8.8	Approximate	7-11 Ma	*Selenoportax giganteus*	Bibi [27]	Barry et al. [28]; Badgley et al. [29]
Stem Hippotragini	6.4	Minimum	6.4–13 Ma	*Saheloryx tchadensis Saheloryx solidus Tchadotragus sudrei*	Geraads et al. [32]	Lebatard et al. [33]
Crown Tragelaphini	5.72	Approximate	4.7–6.7 Ma	*Tragelaphus moroitu Tragelaphus sp. cf. spekii*	Thomas [34]; Haile-Selassie et al. [35]	WoldeGabriel et al. [36]; Deino et al. [37]
Crown Reduncini	5.1	Minimum	5.1-7 Ma	*Redunca ambae*	Haile-Selassie et al. [35]	Renne et al. [38]
Crown Alcelaphini	4.5	Minimum	4.5–7.0 Ma	*Damalacra neanica*	Gentry [39]; Vrba [40]	Hendey [41]
Crown Hippotragini	3.6	Minimum	3.6–6.5 Ma	*Hippotragus sp. Oryx sp.*	Vrba and Gatesy [42]; Gentry [43]	Deino [44]
Stem *Tragelaphus euryceros*	3.4	Minimum	3.4-4.5 Ma	*Tragelaphus rastafari*	Bibi [45]	WoldeGabriel et al. [46]
Stem *Tragelaphus strepsiceros*	3.4	Minimum	3.4–4.5 Ma	*Tragelaphus lockwoodi*	Reed and Bibi [47]	WoldeGabriel et al. [46]
Crown *Kobus ellipsiprymnus + K. leche*	2	Minimum	2.0–3.0 Ma	*Kobus ellipsiprymnus*	Gentry [48]	Feibel et al. [49]
Crown *Connochaetes* spp.	1.15	Minimum	1.15–2.15 Ma	*Connochaetes taurinus*	Gentry and Gentry [50]; Vrba [40]; Gentry [51]	Hay [52]; Deino [53]
Crown *Alcelaphus buselaphus*	0.6	Minimum	0.60–1.0 Ma	*Alcelaphus buselaphus*	Vrba [40]	Clark et al. [54]

Basic information on the 16 fossil calibration points used in this study. Full details of each are given as Additional file 1.

**Figure 2:**
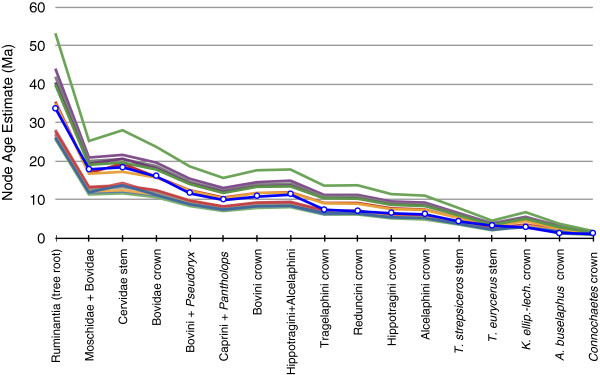
**Median age estimates by node for each of the 16 single-calibrated analyses and the analysis using all 16 fossil calibrations (data in Table**2**), covering the tree root (far left) and the 16 calibrated nodes.** A large range of ages is obtainable with the use of different calibration points. The multi-calibrated analysis (blue with circles) provides estimates that are intermediate in age for older nodes, and towards the younger end of the range for younger nodes (<10 Ma).

The median estimated molecular ages of the different nodes from all the different analyses is shown in Table 2, along with the mean and range of node estimates across all the analyses. Table 2 shows that using only a single calibration point can produce a very wide range of age estimates, with maximum ages being more than double the minimum ages obtainable. In general, older calibration points produced older age estimates, and younger (ingroup) calibration points produced younger age estimates. The use of 16 calibration points results in age estimates intermediate among those of the single-calibration analyses, with older nodes falling toward the mean of the range of estimates, and younger nodes (<10 Ma) falling towards the younger limit of the range of estimates (Figure 2). This may be a result of the age distribution of the 16 calibration points, ten of which are less than 10 Ma in age on the final tree (Figure 1). Node ages in the multicalibrated analysis are also more often associated with smaller error ranges, which was not necessarily to be expected with the use of a larger number of calibration points [55] (Figure 1). Not surprisingly, the multicalibrated tree displays higher rate heterogeneity (in Tracer, ucld.stdev values around 0.22) compared to the single-calibrated analyses (0.14-0.16).

**Table 2:** Node age estimates (Ma)

**Right: nodes below: calibrations**	**Ruminantia (tree root)**	**Moschidae + Bovidae**	**Cervidae stem**	**Bovidae crown**	**Bovini + Pseudoryx**	**Caprini + Pantholops**	**Bovini crown**	**Hippotragini + Alcelaphini**	**Tragelaphini crown**	**Reduncini crown**	**Hippotragini crown**	**Alcelaphini crown**	***T. strepsiceros *stem**	***T. euryceros *stem**	***K. ellip.-leche *crown**	***A. buselaphus *crown**	***Connochaetes *crown**
Fossil age (Ma)	(constrained to 65–25)	23	16.6	ca.18	10.2	8.9	ca.8.8	6.4	ca.5.7	5.1	3.6	ca.5	3.4	3.4	2	0.6	1.15
Probability Median Age (Ma)	45	25.5	21.8	18	12.8	10.8	9	8.8	5.7	6	4.8	5.7	3.9	3.9	2.5	0.8	1.6
Moschidae stem	53.2	**25.2**	28	23.7	18.6	15.6	17.6	17.8	13.6	13.7	11.4	11	7.8	4.5	6.7	3.7	1.8
Cervidae stem	40.4	19.3	**20.6**	18.1	14.2	12	13.4	13.6	10.3	10.3	8.7	8.4	6	3.5	5.1	2.8	1.4
Bovidae crown	39.8	19	19.6	**17.8**	14	11.7	13.3	13.4	10.3	10.2	8.6	8.3	5.9	3.4	5	2.8	1.4
Bovini stem	34.98	16.7	17.2	15.7	**12.3**	10.3	11.7	11.8	9	8.9	7.5	7.3	5.2	3	4.4	2.4	1.2
Caprini stem	35.4	16.9	19.3	15.9	12.4	**10.5**	11.7	11.9	9.1	9.1	7.7	7.4	5.2	3	4.5	2.5	1.2
Bovini crown	27.8	13.2	13.6	12.4	9.7	8.2	**9.2**	9.3	7.1	7.1	6	5.8	4.1	2.4	3.5	1.9	1
Hippotragini stem	28.1	13.3	13.7	12.4	9.7	8.2	9.2	**9.4**	7.2	7.1	6	5.8	4.1	2.4	3.5	2	0.95
Tragelaphini crown	26	11.8	12.1	11.1	8.7	7.3	8.2	8.3	**6.2**	6.4	5.3	5.2	3.7	2.1	3.1	1.7	0.9
Reduncini crown	26.2	12.1	12.4	11.3	8.9	7.4	8.4	8.5	6.5	**6.6**	5.5	5.3	3.8	2.2	3.2	1.8	0.9
Hippotragini crown	26.8	12.4	14.3	11.7	9.2	7.7	8.7	8.9	6.7	6.7	**5.5**	5.5	3.9	2.2	3.3	1.8	0.9
Alcelaphini crown	27.9	13.3	13.6	12.4	9.8	8.2	9.2	9.3	7.2	7.1	6	**5.7**	4.2	2.4	3.5	1.9	1
*T. strepsiceros* stem	27.1	12.8	13.2	12	9.4	7.9	8.9	9	6.9	6.9	5.8	5.6	**4**	2.3	3.4	1.9	0.9
*T. euryceros* stem	44	20.9	21.6	19.6	15.4	13	14.5	14.9	11.2	11.2	9.5	9.2	6.5	**3.8**	5.5	3.1	1.5
*K. ellips.-leche* crown	26.1	11.9	13.7	11.2	8.8	7.4	8.3	8.4	6.5	6.3	5.4	5.2	3.7	2.2	**2.8**	1.7	0.9
*A. buselaphus* crown	25.7	11.3	11.6	10.6	8.3	7	7.8	8	6.1	6.1	5.1	4.8	3.5	2	3	**1.2**	0.8
*Connochaetes* crown	41.9	20	20.5	18.7	14.6	12.3	13.8	14.1	10.7	10.7	9	8.7	6.2	3.6	5.3	2.9	**1.5**
All 16 calibrations	33.7	**17.9**	**18.4**	**16.2**	**11.8**	**10.1**	**11**	**11.5**	**7.4**	**7.1**	**6.6**	**6.3**	**4.4**	**3.3**	**2.9**	**1.3**	**1.3**
Mean	33.2	15.8	16.7	14.8	11.5	9.7	10.9	11.1	8.4	8.3	7.0	6.8	4.8	2.8	4.0	2.2	1.2
Max	53.2	28	25.2	23.7	18.6	17.6	13.6	4.5	7.8	13.7	6.7	17.8	11.4	11	1.8	3.7	15.6
Min	25.7	11.6	11.3	10.6	8.3	7.8	6.1	2	3.5	6.1	2.8	8	5.1	4.8	0.8	1.2	7

Results of the sixteen single-calibration analyses and the one analysis using all 16 calibrations. For each analysis (row), bold text indicates the node(s) used as a calibration point.

## Discussion

### Fossil calibration points: single, few, or many?

Multiple fossil calibrations have already been argued as preferable to single calibrations, particularly given that rates of molecular evolution are not constant across a phylogeny, and that rate variations may not necessarily be autocorrelated among adjacent branches [56-59]. The results here provide further support for the use of multicalibrated analyses and additionally illustrate the very wide range of age estimates that may be obtained when only a single calibration point is used.

Several authors have attempted to selectively choose points from an available pool of fossil calibrations by evaluating the internal congruence of molecular age estimates provided by these calibrations, both against each other and against the fossil record [60-64]. While there may be interesting reasons to use the internal congruence of molecular age estimates from multiple calibration points to identify outliers, such an approach is ultimately a poor substitute for the careful selection of calibration points from the paleontological record [2,6]. It is also potentially circular in reasoning (deriving a calibration point from the fossil record, the estimates from which are then assessed against the fossil record, and these then used to discard a given data point because it apparently doesn’t hold up to the fossil record). Provided a fossil’s age and phylogenetic position have been properly supported as detailed in ref. [2], a low molecular age congruence score should probably not be used to discount it as a molecular rate calibration point. There are numerous reasons why estimates of divergence times derived from molecular data could differ with choice of calibration point, and why molecular and fossil estimates of age might disagree. Among these are rate heterogeneity outside the statistical bounds of the uncorrelated lognormal clock e.g. large population size or generation time changes, [65,66], node-density effects [67], ancestral polymorphism, and time-dependent rate variations that produce artificial branch length compression at older nodes (e.g. saturation) and extension towards the tips [63,68,69]. Ultimately, the use of prior probability distributions and soft bounds for calibration means that the effects of an erroneous fossil calibration within a multiple calibration analysis is limited [70]. Table 2 and Figure 2 show that the use of all 16 calibration points produces a tree that balances aspects of all the calibration points, and is not overly affected by some of the more extremely different points. As might also be expected, the multicalibrated tree based on all 16 calibrations is also more congruent with the predicted ages of the calibrated nodes themselves (lowest sum of percent error of the estimate at each of the 16 nodes with respect to the median age of the probability distribution, Additional file 3: Table S2). Using all available and vetted fossil calibration points therefore seems a better approach than relying on assumptions of molecular clock internal congruence to discard what might be perfectly good information from the fossil record.

### New age estimates for the phylogeny of Bovidae and Ruminantia

Many of the calibration points used separately provide very young ages for the entire phylogeny, including ages for certain nodes that are even younger than the minimum age as established from the fossil record (Table 2). This is an interesting result given that molecular rate estimates are very often found to be older than expectations from the fossil record [71]. Molecular estimates of a node’s age are actually ranges defined by probability distributions, and these can vary greatly depending on the type of genomic data used e.g., [63], the choice and number of calibration points (e.g. this study), choice and number of taxa [72], and choice of the many (ever increasing) parameters of a phylogenetic analysis e.g., [73]. Therefore, the molecular age estimates produced by different analyses should be considered as hypotheses open to further testing by the fossil and genomic records. In the discussion that follows I identify node ages using their 95% probability ranges. I refer extensively to the results of the study by Hassanin et al. [8], which used six calibration points from the artiodactyl tree, with a slightly older crown Bovidae calibration among these. Though I used a pruned version of their same genomic dataset, the analysis methods differ greatly between the two studies. References to ages in the study by Hassanin et al. [8] use the mean ± 2 standard deviation values of their BD-Soft model results. References to ages in the study by Meredith et al. [59] use their DNA Soft IR model.

The new multi-calibrated phylogeny (Figure 1), provides divergence age estimates that are younger overall (~1 to 4 Ma) than those of Hassanin et al. [8], from which the current genomic dataset was derived. These authors used a similar but older crown Bovidae calibration in addition to five other calibrations from other parts of the artiodactyl tree, as well as different software for estimation of divergence times. The largest differences are seen with age estimates towards the base of the tree rather than in the ingroup, which might in part result from the use here of a much reduced taxonomic subset, in addition to a larger number of younger calibrations that produce younger dates (Table 2). Lineage-specific rate variations may also have some effect, as two out of six of Hassanin et al.’s calibration points were close to the origin of Cetacea, which as a clade has been shown to have slow rates of molecular evolution relative to other mammals, potentially inflating estimates of divergence times in other parts of the tree [59]. It is worth noting, however, that age estimates from the analysis using only the crown Bovidae calibration (Table 2) are, with the exception of the root node, not greatly different from the age estimates found by Hassanin et al., indicating that the older dates of their study could be replicated here even with a reduced taxonomic subset and a different calibration scheme.

The multicalibrated tree gives a root age (origin of crown Ruminantia) at 39.3–28.8 Ma (95% range, median 33.7 Ma). This is younger than Hassanin et al.’s [8] age of 51.6 ± 9.8 Ma, and a better match for the 41.4–35.1 Ma estimate by Meredith et al. [59]. These younger estimates at first seem too young given that the fossil record of fossil ‘ruminants’ extends back to the middle Eocene, to around 45 Ma [74]. However, the earliest fossil (probably stem) tragulids are in fact only around 33 Ma in age [74,75]. If Leptomerycidae or Lophiomerycidae are accepted as belonging within crown Ruminantia [9,74,76,77], then that would require the crown clade to be at least 42 Ma in age [74], but these and other fossil ruminant lineages might alternately be found to lie on the stem group. O’Leary et al. [14] also provide support for younger dates. Though they did not sample multiple ruminant taxa, they date the origin of Cetruminantia (ruminants plus hippos and whales) to a minimum divergence of 50 Ma, close to Meredith et al.’s 55 ± 2 Ma, and significantly younger than Hassanin et al.’s estimates of 69.7 ± 9.4 Ma.

The age of origin found here for crown Pecora, and Moschidae in particular, is also young compared to previous sources, including the fossil record. According to my results, crown Pecora originated 23.0–19.4 Ma, and the lineage leading to Moschidae diverged from that leading to Bovidae at 19.3–16.6 Ma. This is a match for Meredith et al.’s [59] estimates of 21.0–18.2 Ma for Pecora and 19.1–16.4 Ma for Bovidae + Moschidae, and significantly younger than Hassanin et al.’s [8] estimates of 27.6 ± 7.6 Ma and 22.4 ± 4.8 Ma. However, if the earliest stem moschids are late Oligocene in age (>23 Ma), this means the origin of crown Pecora must have taken place by this time. The much younger age returned by the analysis is interesting considering that the earliest cervid, moschid, and bovid fossils were used as calibration points, and that, when run separately, these three calibrations provide some of the oldest age estimates among all analyses (Table 2). In contrast, nine of the single-calibrated analyses return very young estimates (<16 Ma median age) for the Moschidae-Bovidae split. While the possibility of a younger (c.16 Ma) origin of crown Bovidae could be considered (below), a 19.3–16.6 Ma split for Moschidae-Bovidae is in direct conflict with our current understanding of the fossil record. Resolving this conflict will not be straightforward. Stretching the branch length leading to Moschidae back into the late Oligocene would create long ghost lineages for the other pecoran families. A more basal phylogenetic placement of Moschidae as the first family to diverge in the pecoran radiation would fit the order of appearance of pecoran families in the fossil record, but is not supported by molecular analyses. An alternate, perhaps more likely, possibility is that the assignment of *Dremotherium* to the stem group of Moschidae is incorrect and that the oldest stem moschids are the early Miocene *Pomelomeryx* or the middle to late Miocene *Micromeryx*[78,79]*.* In this case, the divergence of Moschidae from the remaining Pecora could be as young as the molecular data here indicate. Future phylogenetic analyses, particularly on the fossil record of early moschids, may help resolve the conflict.

An early Miocene age for the origin of Pecora, however, does fit the fossil record of Antilocapridae, Giraffidae, Cervidae, and Bovidae, the oldest (stem) fossils of which appear between about 23 and 16 Ma [80,81]. My estimates for a late Miocene origin of crown Cervidae (12.4–9.0 Ma) match those of Hassanin et al. (10.7 ± 2 Ma) [8] and are slightly older than those of Gilbert et al. (9.6–7.7 Ma) [82]. In separate analyses, Gilbert et al. [82] also obtained much older ages for crown Cervidae, but this result used early Miocene cervids to calibrate the node of origin of crown Cervidae, instead of the origin of the stem group as done here (Additional file 1). A middle to late Miocene origin of crown Cervidae fits the fossil record, in which Miocene species of *Euprox,* which may lie close to the origin of the crown node, are dated to c.14–11 Ma or later [21,24].

The young age found here for the origin of crown Bovidae (17.1–15.3 Ma) also contrasts with the older results of most studies to date, but may better fit the fossil record. The oldest fossil bovid is *Eotragus noyei* from 18.3 Ma in Pakistan [23], but since it is not clear whether this and other *Eotragus* species belong on the stem lineage or in the crown clade of Bovidae, there is no reason to believe that crown bovids must have appeared by 18 Ma (see Additional file 1). The middle Miocene bovids *Pseudoeotragus seegrabensis* and *Eotragus sansaniens* have been proposed as some of the earliest representatives of stem Antilopinae and stem Bovinae, respectively [83,84]. Other early Bovinae or stem Bovinae include Miocene ‘boselaphines’ such as *Kipsigicerus labidotus, Protragocerus* spp., *Helicoportax spp.*, and *Sivaceros spp*, and among early Antilopinae or stem Antilopinae, *Caprotragoides potwaricus*, *Tethytragus spp., Gentrytragus spp.*, and early forms assigned to ‘*Gazella*’ [25,83,85-88]. This pool of taxa indicates that the divergence of Bovinae and Antilopinae probably took place during the middle Miocene. A single site of old age that is well dated and at which several of the above mentioned taxa occur is Fort Ternan in Kenya [86]. The uppermost level bearing mammalian (including bovid) remains in the Fort Ternan “B” beds has an age of 13.9 ± 0.3 Ma [89]. A young (17–15 Ma) age of origin of crown Bovidae is therefore a reasonable hypothesis. An important point here is that the phylogenetic position of the oldest fossil bovid [23] is indeterminate relative to the crown bovid node. Such early bovids may represent stem taxa that predate the last common ancestor of extant bovids, and so therefore should not be used to provide minimum ages for crown Bovidae, as many studies to date have done, therefore potentially overestimating the age of this node.

### Bovid phylogeny: topology of the tree using 16 calibrations

The MCC tree resulting from the analysis using all 16 fossil calibration points (Figure 1) and those arising from the 16 single-calibration analyses and uncalibrated analysis are almost all identical. Trees differing slightly in topology to that in Figure 1 were found with 9 of the 16 single-calibrated runs (stem Moschidae, stem Cervidae, stem Hippotragini, crown Hippotragini, crown Tragelaphini, stem Caprini, crown *Connochaetes*, crown *Kobus*, stem *T. strepsiceros*), as well as with the analysis using no calibrations at all (Additional file 1). The main differences observed in these phylogenies involve a rearrangement at the base of the Pecoran radiation (recovery of an *Antilocapra* + Giraffidae clade and the placement of Cervidae basal to it), and inverted relationships among some of the bovid tribes (Reduncini + *Pelea* closer to Antilopini, and Cephalophini + *Oreotragus* closer to the Caprini + Alcelaphini + Hippotragini clade). Some of these relationships are surrounded by poor support values and therefore probably indicate areas of the tree requiring further investigation. These results also indicate that choice of fossil calibration can produce variations in tree topology.

Despite the use of very different methodological approaches, the trees presented here are similar in topology to the maximum likelihood and Bayesian trees of Hassanin et al. [8]. Specifically, Hassanin et al. used 36 nucleotide partitions, and a supertree approach to combine the results of repeated maximum likelihood analyses on overlapping subsets of the matrix. These authors also performed a Bayesian analysis, but using different software and a different evolutionary model (CAT - GTR + Gamma 4). My tree differs from the Bayesian tree of Hassanin et al. (appendix 5 in ref. [8]) and agrees with their maximum likelihood tree (Figure one in ref. [8]) in three main aspects: 1) a resolved basal branching order of Tragulidae - >*Antilocapra* - > Giraffidae - > Cervidae - > Moschidae (all PP = 1) rather than a 3-way polytomy with *Antilocapra* + Giraffidae, Cervidae, and Moschidae + Bovidae cf. ref. [59]; 2) a sister relationship between Alcelaphini and Hippotragini (PP = 0.84), rather than Alcelaphini and Caprini; 3) placement of Reduncini + *Pelea* as closer to Caprini + Alcelaphini + Hippotragini (PP = 0.71) rather than Antilopini; and 4) placement of Cephalophini + *Oreotragus* as sister to Antilopini (PP = 0.72), rather than basal to Caprini + Alcelaphini + Hippotragini (there are variations on 1, 3, and 4 however, in some of the single-calibration runs mentioned above). Naturally, all these relationships would benefit from further testing, especially at nodes with low posterior probability values (PP <0.9). Topological differences towards the base of the tree especially might also be affected by taxon sampling, as the current study only considers a subset of the taxa analyzed by Hassanin et al..

The current MCC tree (Figure 1) differs from the maximum likelihood tree presented by Hassanin et al. [8] and agrees with their Bayesian tree and also ref. [90] in the placement of the saola (*Pseudoryx*) as the sister taxon to Bovini rather than within Bovini (PP = 1), and also in the placement of the rhebok *(Pelea)* as the sister taxon to Reduncini and not within Reduncini (PP = 1). These are significant results, as they provide support for the monophyly of Bovini and Reduncini as traditionally defined on the basis of major morphological differences, and highlight an area of conflict between morphological and genomic approaches to taxonomic classification.

### Genomic vs morphological taxonomy

Over the last 20 years, bovid taxonomy has received much needed revision and clarification as a result of molecular phylogenetic investigations. However, conflicts between genomic and phenotypic (e.g. morphological, behavioral, ecological) delimitations of species and higher taxa persist in different parts of the bovid tree. Presumably on the basis of low molecular distances (short molecular branch lengths), some workers have proposed expanding the composition of traditionally defined Reduncini, Bovini, and Caprini (formerly Caprinae) to include their nearest sister taxa, the saola (*Pseudoryx*) with Bovini, the rhebok (*Pelea*) with Reduncini, and the chiru (*Pantholops*) with Caprini e.g. Figure 1 in ref. [8]. This is in conflict with traditional classifications that distinguish these tribes from their sister taxa on the basis of large suites of advanced phenotypic characters [91-93]. In all three cases, there is an apparent lack of correlation between the rate and amount of molecular and morphological evolution that has taken place e.g. [94]. Perhaps homoplasy, whether of nucleotide substitutions or as a result of the convergence or reversal of morphological characters, could be further investigated as a potential cause for the discrepancies between molecular and morphological distances at these nodes.

**Figure 3:**
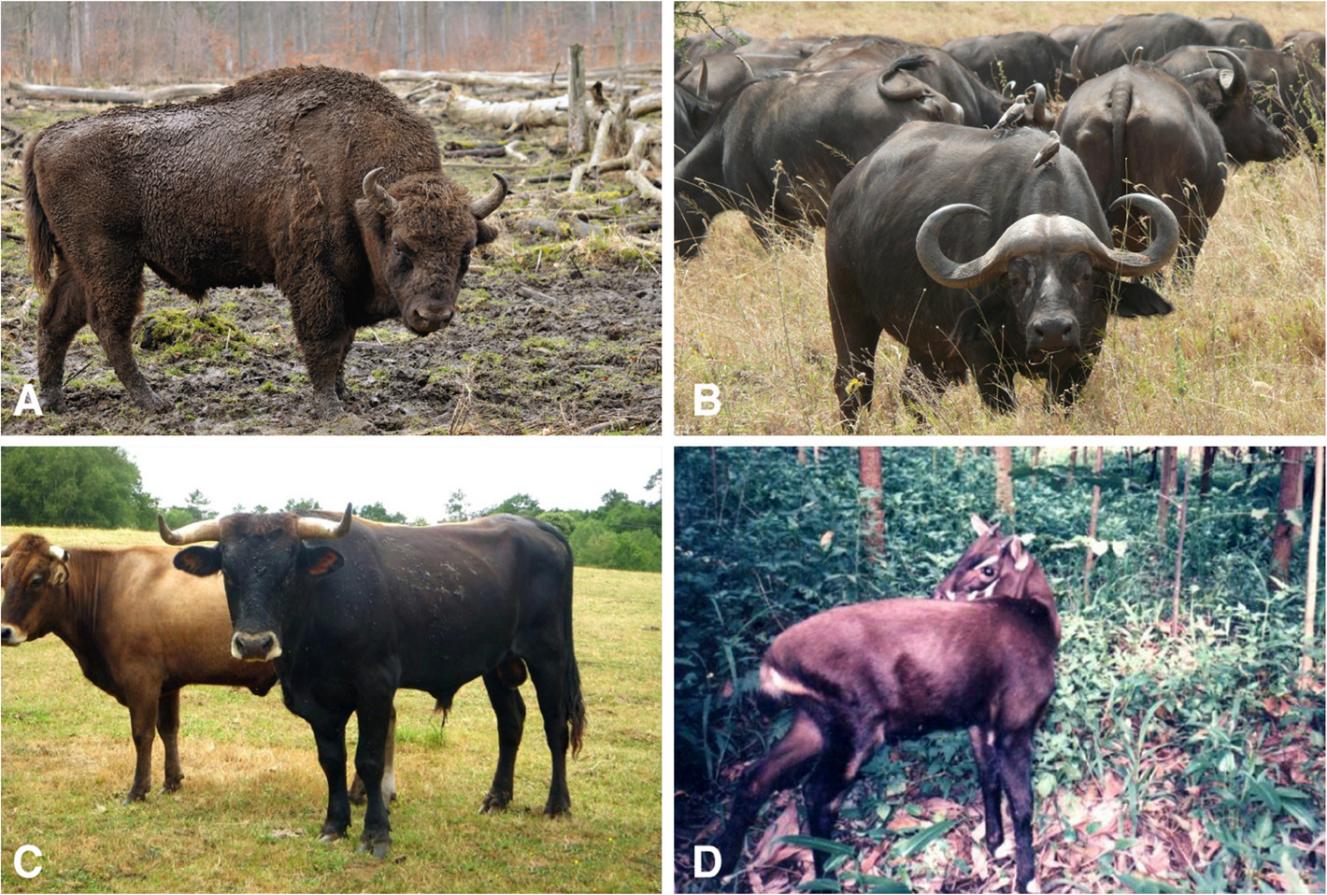
**A large number of morphological, ecological, and behavioral characteristics distinguish Bovini (A-C) from their sister taxon the saola (D, *Pseudoryx nghetinhensis*). A**, European bison (*Bos (Bison) bonasus*), **B**, African buffalo (*Syncerus caffer*), and **C**, domestic cattle (*Bos taurus*). All images from Wikimedia Commons, user credits: Michael Gäbler **(A)**, Haplochromis **(B)**, DFoidl **(C)**, Silviculture **(D)**.

Since there are no agreed upon rules to govern the delimitation of monophyletic higher taxa, a consensus may be difficult to achieve, but at minimum, independent lines of evidence should be considered together. Higher taxa are probably only necessary in so far as they are useful to both specialists and non-specialists alike. In the case of Bovini for example, it is much more practical and intuitive for paleontologists, ecologists, zoologists, and lay people to restrict composition of this clade to the buffaloes and oxen, which are easily recognized bovids of large size with a significant suite of skeletal, dietary, and behavioral adaptations, and broadly similar ecological niches (Figure 3). The saola, in contrast, is a small forest dweller lacking most if not all of the derived features characterizing Bovini, so much so that its skull was found to resemble caprins such as *Capricornis* and *Naemorhedus*[95], but see [96]. The fact that DNA differences between the saola and Bovini are few should probably not be used as the sole criterion for the delimitation of Bovini, otherwise the tribal name risks losing much of its coherency and common utility. Granted, expanding the composition of Bovini to inclide the saola would highlight the surprising genomic similarities among all these animals, but it would do so in spite of large phenotype-level differences, which clearly the genomic sequences alone do not predict. The same case applies to *Pantholops* with respect to Caprini, and *Pelea* with respect to Reduncini. The latter case is particularly worthy of further investigation because though the branch length (and resulting time estimate) between the divergence of *Pelea* and the origin of Reduncini is very short (Figure 1), and the support for reduncin monophyly is poor (PP = 0.71), reduncin fossils of *Kobus porrecticornis* as old as 9.3 Ma are recorded in the Siwaliks of Pakistan [29]. Further study of the Siwalik reduncins with the intent of establishing their relationships to living *Redunca, Kobus,* and *Pelea* could therefore go a long way toward resolving the unstable molecular arrangements at the base of Reduncini.

Conflict is also present with regard to taxonomic revisions made at the species level. Relationships based on mitochondrial genomes benefit from independent confirmation by nuclear genes e.g. [97]. For example, the mitochondrial sister relationships of *Capra sibirica* to *Hemitragus jemlahicus* and *Bos (Bison) bonasus* to *Bos taurus,* which result in the polyphyly of *Capra* and the bisons*,* are contradicted by both nuclear genomic and morphological analyses that both support the monophyly of these two taxa [97-102]. Analyses such as those presented here or by Hassanin et al. [8] similarly support previous findings [103] regarding mitochondrial polyphyly within the bushbuck (here labelled *T. scriptus* 1 and *T. scriptus* 2 + 3 in Figure 1). However, these mitochondrial relationships contradict morphological evidence, and, in light of the examples of the bisons and *Capra*, the elevation of *T. scriptus sylvaticus* to the level of a separate species (*T. sylvaticus*) unrelated to *T. scriptus* should await further confirmation from the nuclear genome [103]. The possibility of incomplete lineage sorting, or mitochondrial lineage introgression among geographically proximate tragelaphin species, as probably occurred within Caprini and Bovini, has not yet been ruled out.

### Neogene diversity dynamics and biogeography of Bovidae

It is tempting to reconstruct the timing and mode of interesting evolutionary, ecological, and biogeographic events on a well-resolved tree of extant taxa. However, doing so in the absence of the fossil record can be highly misleading, as a lineage’s characteristics (e.g. rates of extinction, geographic distribution, habitat preferences) may have changed significantly through the course of its evolution. I provide a few examples of evolutionary hypotheses developed from the dated phylogeny in Figure 1, and compare these to the fossil record.

The bovid subclades (tribes) Bovini, Tragelaphini, Cephalophini, Reduncini, Antilopini, Alcelaphini, Hippotragini, and Caprini comprise some 120 of ~140 living bovids species. Rather than being entirely taxonomic constructs, these bovid tribes comprise large and distinct ecological guilds, each distinguished by suites of unique morphological and behavioral characters that reflect distinct dietary and habitat preferences. These tribal-ecological associations have proved so compelling that paleontologists regularly use the proportions of bovid tribes at fossil sites as an indicator of the range of vegetational habitats found at that site [104-107]. The age of origin of the main ecologically distinct clades (or ‘guilds’) of bovids is therefore of interest from the perspective of the development of modern vegetational biomes, and particularly in Africa, where bovid diversity mostly resides today. According to the dated tree (Figure 1), the origins of Reduncini, Caprini, Alcelaphini, Hippotragini, Cephalophini, and Tragelaphini all took place between 10.1 and 5.4 Ma (maximal 95% interval, or just 9.2–6.3 Ma using median ages), which is a short interval of time considering the 15 to 18 million year history of Bovidae. However in almost all cases, the stem branches leading to these originations are long, stretching back to the middle Miocene, which makes it possible that their late Miocene ‘radiation’ is actually an artifact of differential (non-random) extinction of older lineages. Given that the majority of middle and early late Miocene fossil bovids have not been well studied from a phylogenetic perspective, and given limitations of testing for deep extinction using only extant species, evaluating punctuated (radiation) versus gradualistic evolution (differential extinction) scenarios for the origins of the major ecological subclades (tribes) of Bovidae will benefit greatly from further work on the Miocene fossil record.

The origins of Bovini and Antilopini are older than those of the other bovid tribes (Figure 1). Antilopini is a highly diverse clade that is complexly distributed in terms of geography, such that any single calibrated phylogeny of this clade is full of evolutionary, biogeographic, and ecological hypotheses that could be tested with the fossil record. For example, the springbuck (*Antidorcas*) and the gerenuk *(Litocranius)* are African antilopins adapted to and living in relatively arid habitats. They have totally non-overlapping ranges, the springbuck being restricted to southwestern Africa and the gerenuk to the African Horn. The 8.8–6.4 Ma divergence between these two arid-adapted species might be interpreted as evidence for the late Miocene presence of arid corridors linking the Somali and South West arid zones, much as has been proposed for times in the Pliocene and Pleistocene [108]. The problem with such a scenario is that the restriction of *Antidorcas* to southwestern Africa appears to be a relatively recent phenomenon — the genus is recorded from the late Pliocene of Chad [109] and from the late Pliocene to the Pleistocene in eastern Africa, up until 2.5 Ma or younger in the Afar [110,111], the early Pleistocene in the Turkana Basin [51,112], and the middle Pleistocene in northern Tanzania [50,51]. While no fossil record is known for the gerenuk [51], the wide range of *Antidorcas* throughout most of its history means Pliocene and Miocene phylogeographic inferences based on its modern day geographic range must be made with caution.

In another example, the blackbuck (*Antilope cervicapra*) is historically restricted to southern Asia (Pakistan, Nepal, India, and Bangladesh), but is overwhelmingly bracketed by African species (Figure 1) see also ref. [113]. The age of divergence of the blackbuck lineage from the remaining African Antilopini might therefore be taken to indicate a dispersal event from Africa into southern Asia sometime around 7.4–5.2 Ma. Critically missing from the phylogeny, however, is a large number of spiral-horned antilopin species from the late Miocene Greco-Iranian region, specifically *Prostrepsiceros* spp., from which the blackbuck may be descended [51,114], and which indicate the blackbuck’s origins are probably to be found in Eurasia.

In a similar position, the Reduncini + *Pelea* clade is today entirely African in distribution, and phylogeographic inferences based on a tree of extant taxa might suggest that the origins of the Reduncini are to be found in Africa as well. The fact is, however, that the earliest reduncins known are from Pakistan [25,28,29,115], raising the possibility that the morphological and ecological characteristics of this African clade may have their evolutionary roots in southern Asia, and not Africa.

In Bovini, a deep divergence of African (*Syncerus*) and Asian (*Bubalus*) buffaloes around 7 Ma (8.5–5.7 Ma 95%) might reflect findings from the fossil record. Around 8–7 Ma is the age of hypothesized dispersal of Bovini from southern Asia into Africa in the late Miocene based on the fossil record [26], and 7 Ma is also the age of the oldest Bovini currently known from Africa [116,117]. Furthermore, there is evidence that faunal connections between southern Asia and Africa were stronger in the Miocene than today, becoming progressively weaker over time, and especially by the end of the late Miocene [115,118]. The disruption of gene flow between *Bubalus* and *Syncerus* at around 7 Ma might also be corroborated by evidence for the evolution of *Bubalus* from *Proamphibos* and *Hemibos* in southern Asia, and *Syncerus* from *Ugandax* in Africa since the middle Pliocene or earlier [25,51,119], and may therefore reflect the strengthening of dispersal barriers between what may once have been relatively permeable Afrotropical and Indo-Malayan faunal zones [115,120]. These hypotheses would of course benefit from further paleontological investigation, particularly of the spotty late Miocene African record.

## Conclusions

In a recent paper, Bibi and Vrba [7] proposed that molecular phylogenetic studies of Bovidae could be advanced with the use of better calibration practices. I have here identified sixteen fossil calibration points that can be used as molecular age calibrations or reference points for phylogenetic analyses of Bovidae, Ruminantia, and Artiodactyla. I have shown that the choice of calibration points used in an analysis can significantly affect the resulting molecular age estimates for nodes all across the tree, as well as produce slight variations in topology. As such, incorporation of all vetted calibration data available is argued to be a better approach than using just one or a few calibrations. An analysis of the complete mitochondrial genome using a large number of fossil calibration points here produces younger ages for Bovidae (and Ruminantia), which may be a better fit to the fossil record than the older estimates of previous studies. The resulting tree has also been used to highlight areas of conflict between phylogeographic and evolutionary inferences derived from trees containing only extant taxa and those derived from the fossil record. Further development of nuclear genomic datasets and further incorporation of the fossil record are required to address central questions on rates of genomic versus morphological evolution, gene and species trees, molecular and fossil ages, molecular and morphological differences, and the recognition and timing of ecological radiations and dispersal events in Ruminantia. It is hoped that the inclusion of fossil taxa into a combined morphological-molecular analysis, along with the use of multiple molecular clock calibration points and perhaps different methodological approaches e.g. [73,121] can bring us closer to a more complete evolutionary history of ruminants.

## Methods

I used a subset of the matrix of complete mitochondrial genome matrix presented by Hassanin et al. [8], comprising 112 extant bovids, two moschids, six cervids, three giraffids, one antilocaprid, and two tragulids (these counts include in a few cases more than one individual of the same species). The matrix and analysis parameters were assembled in Beauti v.1.7.4 [122] and the analysis was run in BEAST 1.7.4 [122] using the online computational service Bioportal [123].

The mitochondrial dataset was analyzed as a single partition, using a lognormal relaxed clock with uncorrelated rates [122], the GTR + G + I model as determined by both AIC and BIC criteria in jModelTest 2.1.1 [124,125], with 4 gamma categories, a Yule process speciation tree prior, and empirical base frequencies. A starting tree was used based on that of Hassanin et al.’s Bayesian analysis (Appendix 5 in ref. [8]), pruned down to the 127 taxonomic units used in this analysis, with polytomies arbitrarily resolved, and with branch lengths adjusted to fit prior age distributions (required for BEAST to run with so many priors). An independent analysis of the same dataset but including no calibrations and using a random starting tree was also run to test the effect of a starting tree on the final topology.

The 16 fossil calibrations used are listed in Table 1 and full details on each are given as Additional file 1 following the recommendations of Parham et al. [2]. Of the 16 points, three provide ages for nodes toward the base of the tree, and 13 provide ages for ingroup nodes (i.e. nodes inside Bovidae). These 16 calibrations are by no means exhaustive, and further study and research of the bovid record may find others, and even refine the ones currently used. While covering large parts of the bovid tree, there are conspicuous areas with no calibrations, namely Cephalophini, Antilopini, and Caprini. Cephalophini have nearly no fossil record at all [51]. Antilopini and Caprini on the other hand have a rich fossil record, but there is much ambiguity in the phylogenetic determination of most of the material. Further work should help clarify relationships, especially at the shallower nodes of both of these clades.

Several calibration points were considered but not used because in the current taxonomic sample they would point to the same node as another already used and older calibration point (e.g., stem *Kobus*, a duplicate of crown Reduncini; stem Alcelaphini, a duplicate of stem Hippotragini; stem *Connochaetes*, a duplicate of crown Alcelaphini; stem *Taurotragus*, duplicate of stem *T. strepsiceros*). Other excluded potential calibrations come from the fossil records of bisons, *Capra*, and *Tragelaphus scriptus*, given that all three taxa are found to be polyphyletic on the basis of mitochondrial genomic analysis (but not nuclear genes, discussed in text). In an isolated case, separate analyses (not shown) showed that use of a stem Reduncini calibration produced aberrant results, such as age probability distributions (error bars) much larger than in any other analysis, inclusion of *Pelea* within Reduncini (not found in any other analysis here), and a reversed branching order of Cervidae and *Antilocapra* with the nodes underlying these clades very weakly supported (found in a few other analyses, detailed in text). Including this data point in the multi-calibrated analysis affected the final topology much more so than with other calibration points, and this point was a result excluded.

All calibration points use a probability distribution with soft bounds [126]. The oldest fossil record in a clade provides a minimum age of origination for that clade [3,71]. In cases where the phylogenetic position of the fossil is known with some certainty, a lognormal distribution is used with a 2.5% probability quantile (youngest bound) set at the age of the fossil. If the phylogenetic position of the fossil is not precisely known relative to the clade in question, a normal distribution is used allowing for the actual node age to be equally younger or older than the fossil. Maximal (97.5% probability quantile) node ages are difficult to determine from the fossil record and this setting is to a large degree unavoidably arbitrary. However, the use of multiple calibrations and soft bounds means accurately determining maximal bounds is less crucial than with a single calibration or hard bounds [61,70]. Case-by-case assessments are given in the Additional file 1, but in general I chose conservatively large probability distributions. The use of soft bounds meant the analysis could still date a node to an age older or younger than the 95% probability interval.

No monophyletic constraints were used so as not to constrain tree topology. Tree root height was assigned a uniform distribution between 25 and 65 Ma with an initial starting value of 45. All analyses comprised 2 independent runs of 20,000,000 generations each, sampling every 1000 generations, and burn-in set to 2000 trees (10%) for each run. Examination of the resulting log files in Tracer [122] indicated convergence of all parameters in the independent runs and combined effective sample sizes greater than 95 for all values (and in most cases well above 200). Trees files from each run were combined using LogCombiner [122] and a maximum clade credibility (MCC) tree was created using Tree Annotator [122] with a burn-in of 4000 trees, using median heights and a posterior probability limit of 0.5.

## Availability of supporting data

Full details on the sixteen fossil calibration points are given as Additional file 1 accompanying this manuscript online. The datasets (matrix and trees resulting from the phylogenetic analyses) supporting the results of this article are available in the TreeBase repository, http://purl.org/phylo/treebase/phylows/study/TB2:S14132.

## Competing interests

The author declares that he has no competing interests.

## Supplementary Material

Additional file 1 Supplementary text with details of fossil calibrations used. Click here for file

Additional file 2: Table S1 Molecular divergence age estimates for ruminant clades from the analysis using all 16 fossil calibrations. Click here for file

Additional file 3: Table S2 Percent difference between the molecular estimate and the median fossil age for the root and 16 calibrated nodes. These references are then summed at far right for each tree, and below for each node. Not surprisingly, the tree using all 16 calibrations also provides estimates with the lowest difference from the expected median age. The nodes with the highest summed difference scores are the *Alcelaphus buselaphus* crown and the *Kobus ellipsiprymnus + K. leche* crown nodes. Click here for file
