# Supplementary material for: A multi-calibrated mitochondrial phylogeny of extant Bovidae (Artiodactyla, Ruminantia) and the importance of the fossil record to systematics
Source: BMC Evol Biol. 2013 Aug 8;13:166. doi: 10.1186/1471-2148-13-166 (PMC3751017; doi:10.1186/1471-2148-13-166)
Supplement: Additional file 1 — Supplementary text with details of fossil calibrations used. [file 1471-2148-13-166-S1.pdf]

SUPPLEMENTARY MATERIALS to accompany

A Multi-Calibrated Mitochondrial Phylogeny of Extant Bovidae (Artiodactyla, Ruminantia) and the Importance of the Fossil Record to Systematics

Faysal Bibi

Museum für Naturkunde, Leibniz Institute for Research on Evolution and Biodiversity at the Humboldt University Berlin, Invalidenstrasse 43, Berlin 10115, Germany

Current address: Department of Mammalogy, American Museum of Natural History, 200 Central Park West, New York, NY 10024, USA fbibi@amnh.org

Table of Contents

Link to TreeBase archive	S2
Supplementary Table 1 caption	S2
Fossil Calibration Data	S2–S28
References	S28–S40

As separate files: Supplementary Table 1, Supplementary Table 2

The mitochondrial **matrix** and all maximum clade credibility **trees** resulting from all analyses are available online through TreeBase at the following link: http://purl.org/phylo/treebase/phylows/study/TB2:S14132

Supplementary Table 1. Molecular divergence age estimates for ruminant clades from the analysis using all 16 fossil calibrations.

Supplementary Table 2. Percent difference between the molecular estimate and the median fossil age for the root and 16 calibrated nodes. These differences are then summed at far right for each tree, and below for each node. Not surprisingly, the tree using all 16 calibrations also provides estimates with the lowest difference from the expected median age. The nodes with the highest summed difference scores (i.e. nodes that exhibited the highest proportion of molecular age error) are the *Alcelaphus buselaphus* crown and the *Kobus ellipsiprymnus* + *K. leche* crown nodes.

Fossil Calibration Data

Calibrated nodes are defined and identified with respect to living taxa (crown clades) as molecular phylogenies normally consider only extant or recently-extinct taxa. The phylogenetic relationships of fossil bovid taxa have typically been proposed descriptively, or through phylogenetic analyses that are limited in taxonomic representation and/or number of character. A few notable examples of larger phylogenetic analyses that include fossil taxa are those of Gentry

[1], Geraads [2], Vrba [3], Vrba and Gatesy [4], and Bibi [5]. In selecting the fossil specimens or species representing the earliest known appearances of a clade, I have focused on the most reliable information available through the literature or on my own personal studies of fossil specimens, or both. Poorly documented single occurrences, especially any greatly stretching the first appearance datum of a taxon, were not relied upon. In cases where a first appearance datum is taxonomically sound but based on uncertain provenience (e.g. *Taurotragus* from the Chemeron Formation), I relied on the next-youngest occurrence in addition.

All clade calibration ages given here are minimum or approximate ages and probability distributions extrapolated from these come from my own assessment of the fossil record and the paleontological literature. It will be clear from the data that some calibrations are much better substantiated than others, and the details provided here should help others evaluate (and potentially improve) each calibration point themselves. Some may be used directly for dating, others for comparisons with clade origination ages derived from molecular clocks. All calibration data may be expected to change or be refined with further discovery and improvement of the fossil record and our understanding of it. This list is not exhaustive and many more candidate calibrations can still be added.

All references to Pleistocene refer to the recently redefined Pliocene-Pleistocene boundary at 2.588 Ma (previously at 1.8 Ma) [6]. The term crown clade refers to a node-based clade originating with the last common ancestor of two or more extant species or organisms (e.g. Bovidae) [7]. The stem group, or stem lineage, is the ancestral lineage leading to a crown group (e.g. stem Bovidae). The branch-based clade that comprises a crown clade plus the stem leading to it is the 'total' or 'pan-' clade (e.g. Pan-Bovidae) [7]. Recognition of extant species follows

common accounts [8-11], rather than classifications that promote the elevation of most subspecies to the level of species [e.g. 12].

Fossil calibration data are presented, following the recommendations of Parham et al [13], in the following format:

Clade Name

Definition: a phylogenetic definition of the clade [e.g. 7]

Calibration point: the name and placement of the respective calibration point

Age: Numerical age of the oldest known fossil

Probability distribution type, 2.5–97.5% probability range in Ma (mean (x), standard deviation (sd or log(sd)), and median (M) used to create the required probability distribution 95% range in Beauti v.1.7.4 [14])

1. Identification of species and, when feasible, the best-preserved and oldest specimens on which apomorphies relevant to the diagnosis of the node being dated are visible. In some cases, evidence for cladogenesis relies on contemporaneous occurrences of multiple taxa rather on any individual specimen or collection of specimens (e.g. Pan-Cervidae).
2. Identification of the apomorphies diagnosing the specimen or taxon as belonging within the clade in question. These may be in the form of references to in-depth discussions or treatments (phylogenetic analyses) in other studies.
3. Evidence for clade monophyly and identification of any major issues that may affect reconciliation of morphological and molecular phylogenetic analyses of the clade in question.

4. Identification of locality and stratigraphic information from which the relevant specimen, specimens, or taxa are known to have been collected.
5. Numerical age and dating information for the relevant specimen, specimens, or taxa, from which the clade age above is derived.
6. Justification for the calibration prior probability distribution parameters. A lognormal prior distribution with the upper 2.5% limit set to the fossil's age is used when the oldest fossil belongs to the clade in question. This means that there is a 97.5% chance that the node being calibrated will be older than the fossil. A normal distribution with the mean set to the fossil's age is used in cases where it is not certain whether the fossil belongs inside the clade in question or on its stem lineage. This means that there is a 50-50 chance that the node age will be either older or younger than the fossil. While fossil evidence helps provide a good minimum age for a divergence, maximal ages are much more difficult to assess. The main criterion considered here is the completeness of the fossil record of the lineage in question just prior to the origination of the node in question [13, 15]. I conservatively choose wide intervals.

Pan-Cervidae

Definition: the clade consisting of all extant Cervidae and all species (living or extinct) that share a more recent common ancestor with Cervidae than with any other living ruminant.

Calibration point: stem Cervidae

Minimum age: 16.6 Ma

Lognormal, 16.6–28.4 Ma (x = 22.0 Ma, log(sd) = 0.14, M = 21.8 Ma).

1. *Procervulus praelucidus*, *Ligeromeryx praestans*, *Acteocemas infans*.
2. Gentry [16 and references therein] list some characters allying such early Miocene taxa with Cervidae, including the presence of antlers with a proximal permanent pedicel and an upper deciduous antler. These taxa retain conserved characters such as the absence of an antler burr, which indicates an earlier mode of antler development different from that of the crown clade, meaning they should be considered stem Cervidae [16; G. Rössner pers comm].
3. Cervidae is by all accounts monophyletic though questions have in the past surrounded the relationship of *Hydropotes* to remaining cervids [17]. A revised taxonomy of cervids based on molecular phylogenies is given by Gilbert et al. [18] and Hassanin et al. [19].
4. The three fossil species come from European Land Mammal Zone (MN) 3 in Germany, France, and Spain [16].
5. The upper and lower bounds of MN 3 are not precisely determined, and the age range including uncertainties is given by Agusti et al. [20] as 22.1–16.6 Ma (i.e. the early Miocene).
6. The fact that three stem cervids appear within MN 3 means that the line leading to Cervidae diverged from the remaining Pecora during or before this time. Given a general lack of understanding of the phylogenetic relationships of late Oligocene ruminants relative to the living clades, a lognormal distribution is chosen with its 95% range covering the maximal age range of MN 3 and extending back to the beginning of the Late Oligocene (28.4 Ma).

Pan-Moschidae

Definition: the clade consisting of all extant Moschidae and all species (living or extinct) that share a more recent common ancestor with Moschidae than with any other living ruminant. In the current analysis recognizing extant taxa only, the node from which Pan-Moschidae originates is that defining Moschidae + Bovidae.

Calibration point: stem Moschidae

Minimum age: 23 Ma.

Lognormal, 23.0– 28.4 Ma (x = 25.5Ma, log(sd) = 0.055, M = 25.5 Ma)

1. The oldest fossil moschid is *Dremotherium feignouxi* [16, 17, 21].
2. The affinities of *Dermotherium* with Moschidae appear to be based largely on the presence of a laterally enclosed subcentral tympanohyal on the auditory bulla [17]. This and other characters are described and discussed by Webb and Taylor [22] and Janis and Scott [17, 23], with numerous indications that *Dremotherium* is a stem taxon.
3. Living moschids are represented by a single genus with five or more species [21]. The position of Moschidae in Pecora has a controversial history, with moschids treated as stem cervids, as the sister clade to Cervidae, or as sister to Bovidae, among other configurations [17, 21]. An early molecular analysis found some support for moschids being the sister group of cervids [24], as did a supertree approach [25], but more recent molecular work has placed the clade as the sister taxon to Bovidae [19, 26].
4. *Dremotherium feignouxi* is first recorded from the latest Oligocene, from Paleogene European Land Mammal Age (MP) 29 [16].
5. MP 29 is bracketed between < 24 and > 23 Ma [27 fig. 20.4].

6. The divergence of the lineage leading to Moschidae from the remaining Pecora appeared before the Oligocene-Miocene boundary at 23 Ma. Given uncertainty about moschid origins just prior to this time, a lognormal distribution with a 95% interval spanning the Late Oligocene (23–28.4 Ma) is considered appropriate.

Crown **Bovidae**

Definition: the clade originating from the last common ancestor of *Bos taurus* and *Gazella dorcas*.

Calibration point: crown Bovidae

Age: Around 18 Ma

Normal, 16–20 Ma (x = 18, sd = 1, M = 18)

1. Specimens of *Eotragus noyei* as published by Solounias et al. [28].
2. The affinities of *Eotragus noyei* to Bovidae are established mainly by its possession of permanent and unbranched horn cores, which presumably also carried unbranched keratinous horn sheaths that were not shed annually [23, 28]. The presence of a permanent and unbranched horn core, however, cannot be used to differentiate the earliest members of crown Bovidae from species on the stem lineage. Certain *Eotragus* species have been proposed as members of Bovinae [28-30], and therefore crown bovids, but in my opinion this has not yet been sufficiently established [31]. Gentry et al. [16] and Janis and Scott [17] identify numerous features distinguishing extant bovids from the other pecoran families, but also point

out the difficulties of identifying these character states in early fossils, as well as the uncertainty of character polarity determination.

3. The monophyly of crown Bovidae relative to other ruminant families is firmly established. A lack of understanding of the morphological character transitions underlying the origin of Bovidae means that the exact phylogenetic position of numerous early, conservative species is currently difficult to determine. Molecular analyses find consistent support for a basal differentiation of living bovids into two subclades, Bovinae and Antilopinae [19, 32-36]. This same division has also been proposed on morphological and behavioral grounds [37, 38].
4. *Eotragus noyei* is the most securely dated of several early Miocene candidate bovids or stem bovids of similar age (including the contemporaneous or older *E. minus* [39, 40]). The holotype and all referred specimens of *E. noyei* come from sites GSP-Y 747 and GSP-Y 846, from the Kamlial Formation of northern Pakistan [28].
5. The Kamlial Formation is dated using magnetostratigraphy to between 18.3 and 14.3 Ma [41], and the localities from which *E. noyei* derive are dated to between 18.3 and 18.0 [28], presumably also on the basis of magnetostratigraphy.
6. A large number of calibrated molecular studies to date have used the age of *Eotragus noyei*, as the age of origin of crown Bovidae. However, since *Eotragus noyei* may be either a crown or stem bovid, and given a decent late Oligocene to early Miocene record showing an absence of bovids or definite stem bovids and a relatively sudden appearance of *Eotragus* in Asia and western Europe at around 18-17 Ma [16], it is reasonable to assume that the oldest *Eotragus* probably lies within a few million years, either way, to the last common ancestor of all living

Bovidae. A normal distribution which allows the crown clade to have originated within 2 Ma before or after *Eotragus noyei* leaves a broad range for uncertainty.

Note that the minimum date of 18.3 Ma for the origin of crown Bovidae given by Benton and Donoghue [42] was based on the attribution of *Eotragus* to 'Boselaphini' in the paleontological literature. 'Boselaphini' is known to paleontologists to be a paraphyletic ancestral group diagnosed essentially by plesiomorphic characters, and is not equivalent to crown Boselaphini (defined by the most common recent ancestor of the nilgai and chousinga). Most references to Boselaphini with regards to Miocene fossils have referred to primitive bovids or primitive Bovinae, and not necessarily members of crown Boselaphini (or even crown Bovinae). See Bibi et al [ref. 31 fig. 1].

Pan-Bovini

Definition: the clade consisting of Bovini and all species (living or extinct) that share a more recent common ancestor with Bovini than with *Pseudoryx nghetinhensis*. In the current analysis recognizing extant taxa only, the node from which stem Bovini originates is equivalent to that defining Bovini + *Pseudoryx*.

Calibration point: stem Bovini

Age: 10.2 Ma minimum

Lognormal, 10.2-16 Ma (x = 12.9, log(sd) = 0.1115, M = 12.8 Ma)

1. *Selenoportax vexillarius* is the oldest bovid species to display derived morphological features associating it with the early evolution of Bovini [5, 43]. It and its presumed descendent *S. lydekkeri* (alternately called *S. falconeri*) are recorded in the Siwaliks from 10.2 to 8.9 Ma [44, 45].
2. The relationship of late Miocene 'boselaphines' such as *Selenoportax* to Bovini had been proposed since Pilgrim [46] and were more recently addressed by Bibi [5, 43]. *Selenoportax vexillarius* and *S. lydekkeri* share with Bovini synapomorphies of large size, a broad skull, and divergent horns. A preliminary phylogenetic analysis of cranial characters [5] provided some support for placing *Selenoportax* spp. among stem Bovini. My observations on as yet unpublished material (Gentry, Solounias, Barry in prep.) suggest that dental characters diagnostic of crown Bovini—complication of occlusal enamel surfaces, tall and wide basal pillars (entostyles and ectostylids) involved in occlusion, and possibly increased crown height —are not well developed in either species.
3. Discovery of the living saola (*Pseudoryx nghetinhensis*) [47] has brought up its relationships to Bovini and to the nature of the last common ancestor of Bovini. Maximum likelihood and parsimony analyses of mitochondrial genomes nested the saola (*Pseudoryx nghetinhensis*) within Bovini, as a sister clade to Bovina (*Bos* + *Bison*) [19, 48, 49]. The saola appears to possess none of the major morphological apomorphies of Bovini [e.g. 50, but see 51], and so its placement inside the clade would require a massive amount of evolutionary convergence to have occurred in Bovina and Bubalina (or massive reversal in the saola). However, bayesian analyses of the same datasets [19, appendix, 48] as well as a recent nuclear phylogeny [52]

place the saola as the sister taxon to Bovini, a much more plausible hypothesis from a morphological and ecological point of view.

4. *Selenoportax vexillarius* is recorded in the Siwaliks of Pakistan, from sites that have been precisely dated using magnetostratigraphy [44, 45].
5. The first appearance record of *Selenoportax vexillarius* in the Siwaliks is at 10.2 Ma [45].
6. Though primitive for Bovini, *S. vexillarius* is already quite derived compared to other contemporaneous 'boselaphines' such as *Miotragocerus, Tragoportax*, and *Sivaceros*, and relative to the living *Pseudoryx*. The ancestry of *Selenoportax* may lie with the mid-Miocene *Helicoportax* [53], and the last common ancestor of Bovini and the saola could be older still. Therefore I chose a lognormal distribution with its 2.5% upper limit set to 10.2 Ma and a 97.5% lower limit spanning back to 16Ma, being shortly after the age of appearance of *Eotragus*.

Crown Bovini

Definition: the clade originating from the last common ancestor of *Bos taurus* and *Syncerus caffer*.

Calibration point: crown Bovini

Age: around 8.8 Ma

Normal, 7-11 Ma (x = 9 Ma, sd = 1, M = 9 Ma)

1. *Selenoportax giganteus* [5, 45, 54].

2. *Selenoportax giganteus* is derived on *S. vexillarius* and *S. falconeri* in: larger size, strong molar ribs, more convoluted enamel, larger m3 hypoconulid with more anteroposterior orientation and with distal flange present; perhaps greater horn core divergence, greater hypsodonty, and the presence of a small horn core pedicel sinus [5, 54]. While still relatively conserved in many features, *S. giganteus* is the oldest fossil bovid that exhibits a basic suite of characters synapomorphic for Bovini. It therefore probably lies close to the last common ancestor of Bovini, but may itself be either on the stem lineage or within the crown clade.
3. As for *S. vexillarius* above.
4. *Selenoportax giganteus* is recorded in the Siwaliks of Pakistan [44, 45].
5. The first appearance record of *Selenoportax giganteus* in the Siwaliks is at 8.8 Ma [45].
6. Given uncertainty about the stem or crown position of *S. giganteus*, and given the poor phylogenetic understanding of other fossil Bovini from the late Miocene, I choose a normal distribution centered at 9 Ma, with a wide (± 2 Ma) 95% interval.

Crown Tragelaphini

Definition: the clade originating from the last common ancestor of *Tragelaphus scriptus* and all living bovids more closely related to it than to *Boselaphus tragocamelus* or *Bos primigenius*.

Calibration point: crown Tragelaphini

Minimum age: 5.72 Ma

Normal, 4.7–6.7 Ma (x = 5.7 Ma, sd = 0.5, M = 5.7 Ma)

1. *Tragelaphus moroitu* from latest Miocene Ethiopia, best represented by holotype specimen ALA-VP-2/2 [55]. *Tragelaphus* sp. from latest Miocene of Kenya, originally described by Thomas [56] as *T.* cf. *spekii,* and best represented by specimen KNM LU-852.
2. *Tragelaphus moroitu* and the Lukeino *Tragelaphus* sp. (which are probably conspecific) bear all the synapomorphies of Tragelaphini, including horn cores arising upright, spiralling 270º (i.e. ¾ of a whorl), with three keels and a triangular cross-section that is fairly consistent throughout the horn core, with only weak anteroposterior compression basally if at all; teeth that are simple in morphology, mesodont, and effectively little changed from the middle Miocene 'boselaphine' condition, with relatively long premolar rows (about 60–75% of molar row length). These early tragelaphins, however, do not have any identifiable autapomorphies, nor any apomorphies uniting them with any one living tragelaphin lineage. Instead, they represent a good candidate for the ancestry of all later tragelaphins.
3. The monophyly of Tragelaphini is established on both morphological [5, 57, 58] and molecular [19, 59] grounds.
4. Specimens of the Lukeino *Tragelaphus*, LU-852 are from unspecified levels in the Lukeino Formation [56]. *Tragelaphus moroitu* is recorded from the Asa Koma Member of the Adu Asa Formation [55].
5. The Lukeino Formation has a maximum 40Ar/39Ar age of 6.56 ± 0.05 Ma and is overlain by a basalt dated to 5.72 ± 0.05 Ma [60]. The Asa Koma Member is 40Ar/39Ar bracketed to between 5.54 and 5.77 Ma [61].
6. Whether these earliest tragelaphins were actually crown or stem taxa is not clear, but their morphology suggests they must be close, either way, to the common ancestor of the crown

clade. A few rare and isolated dental specimens from Mpesida and the Lower Nawata (both > 6.5 Ma) have been referred to Tragelaphini [56, 62], but these are not conclusive. A normal distribution with a mean at 5.7 Ma and 95% range covering ± 1 Ma leaves opportunity for a latest Miocene to earliest Pliocene origin of the crown clade.

Pheraios chryssomalos was described as a stem tragelaphin from the late Miocene of Greece [57]. It lacks the synapomorphic characters listed above for Tragelaphini. A wider analysis with a larger number of taxa would help further investigate the intriguing hypothesis that the ancestor of Tragelaphini migrated to Africa from Eurasia during the late Miocene.

Pan-*Tragelaphus euryceros*

Definition: the clade consisting of *Tragelaphus euryceros* and all species that share a more recent common ancestor with it than with any other living bovid species.

Calibration point: stem *Tragelaphus eurycerus*

Minimum age: 3.4 Ma

Lognormal, 4.5–3.4 Ma (x = 3.9, log(sd) = 0.07, M = 3.9)

1. *Tragelaphus rastafari* from the late Pliocene of Ethiopia is a good ancestral candidate for the living bongo [63].
2. Arambourg [64], Gentry [65], and Harris [66] had noted resemblances of the living bongo *(Tragelaphus eurycerus)* to *Tragelaphus nakuae* from the African Plio-Pleistocene. Bibi [63] established *Tragelaphus rastafari* to accommodate older specimens previously assigned to *T.*

nakuae, and discussed the cranial characters relating these two consecutive fossil species to the bongo. *Tragelaphus saraitu* is slightly older and may itself be ancestral to *T. rastafari* [67].

3. No good assessment of the relationships among living tragelaphin species exists based on the fossil record. Mitochondrial and nuclear DNA phylogenies indicate the sister taxon to *T. euryceros* is the sitatunga, *T. spekii* [19, 59]. There is no fossil record known for *T. spekii*.
4. The oldest records of *Tragelaphus rastafari* come from the Lokochot Member of the Koobi Fora Formation, the Basal Member of the Hadar Formation, and site WEE-VP-5 in the Middle Awash [63], all of which have minimum ages provided by the overlying Sidi Hakoma (= Tulu Bor) Tuff.
5. Radiometric dating of the Sidi Hakoma Tuff provides an age of 3.446 ± 0.041 Ma [68].
6. The divergence of the bongo's lineage from those of the other living tragelaphins must have taken place before 3.4 Ma, however probably not much before 4 Ma as *Tragelaphus* cf. *moroitu* from Aramis (4.4 Ma) and *T. kyaloi* from Kanapoi (4.1 Ma) are still relatively conservative in morphology. A 95% maximum range at 4.5 Ma incorporates this uncertainty.

Pan-*Tragelaphus strepsiceros*

Definition: the clade consisting of *Tragelaphus strepsiceros* and all species that share a more recent common ancestor with it than with any other living bovid species.

Calibration point: stem *Tragelaphus strepsiceros*

Age: 3.40 Ma minimum

Lognormal, 3.4–4.5 Ma (x = 3.9 Ma, log(sd) = 0.07, M = 3.9 Ma)

1. *Tragelaphus lockwoodi* [69], from Hadar, Ethiopia.
2. Reed & Bibi [69] argued that numerous apomorphies (e.g. the mediolateral compression of the horn cores along with the loose but high degree of torsion and developed anterior keel, combined with large size) make*T. lockwoodi* a viable candidate for the ancestry of the living greater kudu *(T. strepsiceros)*.
3. No good assessment of the relationships among living tragelaphin species exists based on the fossil record. Molecular phylogenies variably place the greater kudu as sister to the elands (*T. derbianus, T. oryx*) or sister to a larger *T. buxtoni* + *T. scriptus* + *T. euryceros* + *T. spekii* clade [19, 59].
4. *Tragelaphus lockwoodi* has been recovered from the Basal Member / Sidi Hakoma Tuff through to unit SH3 of the Hadar Formation [69].
5. Radiometric dating (40Ar/39Ar) of the Sidi Hakoma Tuff provides an age of 3.446 ± 0.041 Ma [68].
6. Given that the roots of the greater kudu's stem lineage among living tragelaphins must be older than 3.4 Ma, and given the relatively conservative morphology of *Tragelaphus* cf. *moroitu* from Aramis (4.4 Ma) and *T. kyaloi* from Kanapoi (4.1 Ma), a lognormal distribution is chosen with 95% range covering 3.4–4.5 Ma.

Crown Reduncini

Definition: the clade originating with the last common ancestor of *Redunca redunca* and *Kobus kob.*

Calibration point: crown Reduncini

Minimum age: 5.1 Ma

Lognormal 7–5.1 Ma (x = 6.0, log(sd) = 0.08, M = 6.0)

1. *Redunca ambae* [55] from the Middle Awash Kuseralee Member represents what is probably the securest first record of *Redunca*, and therefore of a crown reduncin. Contemporaneous *Kobus subdolus* from Langebaanweg (Gentry, 1980) could be a *Redunca* as well [55, 70]. Earlier fossils of Reduncini in Africa and Asia are referred to *Kobus*, but there is the possibility that these may belong in the stem group. I therefore take the first appearance of *Redunca* as a more secure indication of the origin of the crown group. The appearance of *Redunca* at 5.1 Ma indicates the that the basal divergence between it and *Kobus*, and hence the origin of crown Reduncini, must have taken place by this time.
2. See Haile-Selassie et al. [55] for apomorphies uniting *Redunca ambae* with *Redunca* spp.
3. An important part of the evolutionary history of Reduncini took place in southern Asia, as evidenced by the fossil collections from the Siwaliks [46], but molecular phylogenies can only address the living African taxa. Reduncin monophyly and a sister-clade relationship between Reduncini and *Pelea capreolus* appear well-established by both molecular and morphological-behavioral phylogenetic analyses [19 supplementary fig. 5, 32, 36, 38, 71, 72], though a recent analysis placed *Pelea* inside Reduncini [19 fig. 1].
4. The holotype cranium of *Redunca ambae* (AME-VP-1/42) comes from the Kuseralee Member of the Sagantole Formation, Middle Awash, Ethiopia.
5. The Kuseralee Member is 40Ar/39Ar dated to between 5.55±0.09 Ma and 5.18 ± 0.07 Ma [73].

6. A lack of resolution and understanding of the phylogenetic position of late Miocene reduncins attributed to *Kobus* relative to the crown clade, presents the possibility for the actual origin of the crown reduncins to have taken place earlier in the late Miocene. Therefore a lognormal distribution is chosen with a 95% maximal bound extending a couple of Ma older than the age of *Redunca ambae*.

Crown *Kobus ellipsiprymnus* + *Kobus leche*

Definition: the clade originating with the last common ancestor of *Kobus ellipsiprymnus* and *Kobus leche.*

Calibration point: crown *Kobus ellipsiprymnus* + *Kobus leche*

Minimum age: 2.0 Ma

Lognormal, 2.0–3.0 Ma (x = 2.5, log(sd) = 0.1, M = 2.5)

1. Oldest fossils of *Kobus ellipsiprymnus* are known from Member G of the Shungura Formation, and from shortly thereafter at Olduvai Bed I [58, 65].
2. See Gentry [65] for a description of the Shungura fossil specimens and their identification as *K. ellipsiprymnus.*
3. Morphological analyses support a clade uniting the waterbuck *K. ellipsiprymnus* (including *K. e. defassa*) with the leches, *K. leche* + *K. megaceros*, to the exclusion of *K. kob* and *K. vardoni* [25, 71]. Birungi et al.'s [72] analyses of cytochrome *b,* however, did not support this arrangement, presenting the possibility that the clade uniting the waterbuck and lechwes might have to include the kob and puku.

4. Though a single specimen of *K. ellipsiprymnus* is reported from lower Member G (unit G1), three specimens are recorded from upper Member G (above Unit G13) [65].
5. Shungura Formation Member G Unit 13 is dated to between 2.04 and 2.00 Ma using magnetostratigraphy calibrated with radiometric ages [74].
6. Since the fossil age here is a minimum for the actual node age, and since both extant species are also hypothesized to descend from the late Pliocene *Kobus oricornus* [70], a lognormal distribution is chosen with it's older bound extending back to 3 Ma.

Pan-Hippotragini

Definition: the clade consisting of *Hippotragus equinus* and all species (living or extinct) that share a more recent common ancestor with it than with *Alcelaphus buselaphus*. In the current analysis recognizing extant taxa only, the node from which Pan-Hippotragini originates is equivalent to that defining Hippotragini + Alcelaphini.

Calibration point: stem Hippotragini

Minimum age: 6.4 Ma

Lognormal, 6.4–13 Ma (x = 9 Ma, log(sd) = 0.2, M = 8.8 Ma)

1. *Saheloryx tchadensis, S. solidus*, and *Tchadotragus sudrei* are stem hippotragins from late Miocene Chad [75].
2. Geraads et al. [75] described *Saheloryx tchadensis*, *S. solidus*, and *Tchadotragus sudrei*, providing detailed morphological comparisons and a genus-level phylogenetic analysis indicating these taxa are stem hippotragines.

3. Recent molecular analyses have consistently recovered a sister group relationship between monophyletic Alcelaphini and Hippotragini. The presence of stem hippotragins and early alcelaphins (stem or crown) [62] in the late Miocene therefore provides a minimum date for origination of the Alcelaphini + Hippotragini clade.
4. *Saheloryx tchadensis, S. solidus*, and *Tchadotragus sudrei* derive from the anthracotheriid unit of numerous sites in the area of Toros-Menalla in Chad [75].
5. The anthracotheriid unit has been biochronologically correlated to between 7 and 6 Ma [76]. Using cosmogenic nuclides (10Be/9Be), Lebatard et al. [77] dated the unit to between 7.24 ± 0.38 Ma (at site TM 254) and 6.83 ± 0.45 Ma (TM 266). I chose the latter, younger, age estimate including its error as the minimum date.
6. Little is known about the origins of the Hippotragini + Alcelaphini clade. Taxa including *Pachytragus*, *Protoryx*, *Tethytragus*, *Caprotragoides*, and *Gentrytragus* have been proposed as either stem hippotragini, stem Caprini, or may alternately belong around the origin of the Hippotragini + Alcelaphini + Caprini clade [31, 78-80]. While it is conceivable that Pan-Hippotragini may have originated just earlier than the appearance of the Toros Menalla species, an origin extending back to the beginning of the late Miocene, or earlier, remains a possibility.

Crown **Hippotragini**

Definition: The clade originating with the last common ancestor of *Hippotragus equinus*, *Oryx gazella,* and *Addax nasomaculatus*.

Calibration point: crown Hippotragini

Minimum age: 3.61 Ma

Lognormal 3.6–6.5 Ma (x = 4.9, log(sd) = 0.15, M = 4.8)

1. The oldest fossils that can be securely assumed to belong to crown Hippotragini are specimens of *Hippotragus* sp. and *Oryx (=Praedamalis) deturi,* both from mid-Pliocene Tanzania [81].
2. Apomorphies establishing the associations of these fossil specimens to Hippotragini and to *Hippotragus* and *Oryx* are presented by Gentry [81] and Vrba and Gatesy [4]. Though post-dating the divergence of *Hippotragus* from *Oryx*, these fossil species probably predate the origins of crown *Hippotragus* and crown *Oryx* clades.
3. Monophyly of Hippotragini is supported on both morphological and molecular grounds.
4. Recorded from the Upper Laetolil Beds, Tanzania [81].
5. A minimum age of 3.627 ± 0.018 Ma is based on $^{40}Ar/^{39}Ar$ dating of the Yellow Marker Tuff which overlies the main fossil finds of the Upper Laetolil Beds [82].
6. Given the general rarity and poor resolution of the early Pliocene hippotragin fossil record, the actual age of origin of the crown group could be significantly older than that of the Upper Laetolil fossils. Two specimens from the late Miocene (<6.5 Ma) of Kenya were tentatively attributed to '*Praedamlis?'* (i.e. an *Oryx* relative) by Harris [62]. A wide lognormal age distribution is chosen, with a 95% range covering 3.6 to 6.5 Ma.

Crown Alcelaphini

Definition: The clade originating with the last common ancestor of *Alcelaphus buselaphus*, *Connochaetes gnou*, *Damaliscus pygargus*, and *Beatragus hunteri*.

Calibration point: crown Alcelaphini

Minimum age: 4.5Ma

Lognormal 4.5–7.0 Ma (x = 5.7 Ma, log(sd) = 0.11, M = 5.7)

1. The oldest alcelaphin to be attributed to the crown group is *Damalacra neanica* from the earliest Pliocene of South Africa [83].
2. In describing the species, Gentry [83] justified affinity to Alcelaphini by reference to numerous cranial and dental characters. A morphological phylogenetic analysis by Vrba [3] established the position of this fossil taxon (along with *D. acalla*) within the crown clade and as sister to *Beatragus* spp. A slightly modified version of Vrba's analysis was run by Faith et al. [84], supporting the position of this species (but not that of *D. acalla*) as the oldest known crown alcelaphin.
3. The monophyly of Alcelaphini is firmly established by both morphological and molecular analyses. Vrba's [3] analysis indicates that all known Pliocene and Pleistocene fossil alcelaphins belong within the crown clade. Faith et al.'s [84] re-analysis suggests instead that Pliocene and Pleistocene *Damalacra acalla* and *Parmularius* spp. are stem taxa. Harris [62] described specimens from the late Miocene that might also be stem alcelaphins.
4. The holotype and most specimens of *Damalacra neanica* derive from bed 3aS of the Pelletal Phosphorite Member (PPM) of the Varswater Formation at 'E' Quarry at the site of Langebaanweg [83].
5. Fossils from the PPM at Langebaanweg have been dated to an age of around 5 Ma using biochronological correlation and geological correlation to an earliest Pliocene (post-Messinian) marine trangression [85].

6. Though the age of Langebaanweg is normally taken to be around 5 Ma, given the relative dating used at the site, I extend the minimum 2.5% bound to 4.5 Ma. The fossil record of Alcelaphini older than 5 Ma is very poor. Allowing for the actual origin of crown Alcelaphini to be significantly older than *Damalacra neanica*, I extend the maximum 97.5% bound to 7 Ma.

Crown *Alcelaphus buselaphus*

Definition: The clade originating with the last common ancestor of all extant subspecies of *Alcelaphus buselaphus* (including *A. b. lichtensteini*).

Calibration point: crown *Alcelaphus buselaphus*

Approximate minimum age: 0.6Ma

Lognormal, 0.60–1.0Ma (x = 0.8 Ma, log(sd) = 0.14, M = 0.8 Ma)

1. Specimen BOD-VP-1/20, cranium of *Alcelaphus buselaphus* from the Middle Awash, Ethiopia [3].
2. For apomorphies of *Alcelaphus buselaphus* and the description of the BOD-VP-1/20 skull, see Vrba [3]. This skull is most similar to the living *tora, swaynei, cokii* subspecies and so most likely lies inside the crown clade or else very close to the last common ancestor [3].
3. *Alcelaphus buselaphus* is a widespread and highly variable species. Classifications recognize several subspecies, and some taxonomists have advocated species and even genus level distinction for *A. b. lichtensteini* (as *Sigmoceros lichtensteini*), but molecular phylogenies support subspecific designation [86, 87].

4. BOD-VP-1/20 derives from Bodo locality 1, from sedimentary unit ‘u-t’ of the Middle Pleistocene deposits of the Middle Awash, Ethiopia [88].
5. On the basis of sedimentological, structural, faunal, and archaeological evidence, unit ‘u-t’ at Bodo correlates to just above unit ‘u’ at nearby Dawaitoli and Hargufia, where a maximum age of 0.064 ± 0.03 Ma is indicated by 40Ar/39Ar dating of an underlying tuff [88].
6. The Bodo skull represents the oldest known *Alcelaphus buselaphus*, and the oldest *Alcelaphus* at all (crown or stem) from a middle and early Pleistocene African record rich in alcelaphins. The ancestry of *Alcelaphus* has been linked to *Numidocapra (=Rabaticerus) arambourgi* [3, 89] which is only slightly older if at all [58], and *Numidocapra crassicornis* [3] which is about 1.5–1.0 Ma in age [58]. The genus is therefore unlikely to be older than 1 Ma. Gentry [90] described specimens that appear attributable to *Alcelaphus buselaphus lichtensteini* from the middle Pleistocene of Zaire, or at about 0.5–0.3 Ma [3], potentially supporting a ≥ 0.6 Ma age for the origin of the species.

Crown *Connochaetes* spp.

Definition: The clade originating with the last common ancestor of *Connochaetes taurinus* and *Connochaetes gnou*.

Calibration point: crown *Connochaetes gnou + C. taurinus*

Minimum age: 1.15 Ma

Lognormal, 1.15–2.15 Ma (x = 1.6 Ma, log(sd) = 0.155, M = 1.6 Ma)

1. The earliest appearance of the crown clade is marked by the oldest fossils assigned to the extant species *Connochaetes taurinus* in Olduvai Bed II [58, 89]. The extinct *Connochaetes africanus* is also known from Olduvai Bed II [58, 89] and is also believed to belong within the crown clade [3].
2. See Harris [66, 91] and Gentry and Gentry [89] for descriptions of early fossil *Connochaetes taurinus*. See Harris [66], Vrba [3], and Gentry [58] for description and analysis of characters diagnosing *Connochaetes* and proposing *C. gentryi* as a stem taxon and possible ancestor for both living wildebeest species.
3. *Connochaetes* is by all accounts monophyletic [3, 86].
4. The oldest specimens of *Connochaetes taurinus* are known from Olduvai Bed II [58, 89].
5. Bed II at Olduvai ranges in age from 1.79 to 1.15 Ma [92, 93].
6. The early Pleistocene *Connochaetes gentryi* [66], a stem taxon and a likely ancestor for the crown clade [3], is first known from Olduvai Bed I, the Kaitio Member, and the Upper Burgi Member [58, 66, 91], with a maximum age of around 2 Ma [92]. (Vrba [3] also includes two partial horn cores from the Upper Lomekwi, ca. 2.5 Ma, described by Harris [91] as *Connochaetes* sp., in this species.) The maximum age of origin of crown *Connochaetes* is therefore not likely to be older than 2 Ma.

Pan-Caprini

Definition the clade consisting of *Capra ibex* and all species that share a more recent common ancestor with it than with *Pantholops hodgsonii*.

Calibration point: stem Caprini

Minimum age: 8.9 Ma

Lognormal, 8.9–12.9 Ma (x = 10.8 Ma, log(sd) = 0.095, M = 10.8 Ma)

1. *Aragoral mudejar* from MN10 Spain [94].
2. Alcala et al. [94] diagnose *Aragoral mudejar* as a member of Caprini (= Caprinae) on the basis of large size, moderate hypsodonty, reduced premolar rows, horn cores with relatively large bases, frontal sinuses reaching the horn core base, and a greatly shortened and wide metacarpal. The presence of simple frontal sinuses in the pedicel and horn core base is probably a synapomoprhy of the Caprini + Alcelaphini + Hippotragini clade, with further developments in lineages such as *Capra* and *Ovis*. A greatly shortened and wide metacarpal is a synapomorphy of Caprini [e.g. 38], and therefore helps place *Aragoral* on the line to Caprini, or possibly within the crown clade.
3. Caprini is by all account monophyletic, though its internal subclades have received numerous systematic shakeups [95-98]. *Pantholops* is an enigmatic taxon that has never adequately fit into any of the main bovid tribes. Recent molecular and morphological analyses have placed it as the sister taxon to Caprini, from which it differs in many important behavioral and morphological characteristics [38]. Among these are shortened metacarpals, a character present in *Aragoral mudejar* and Caprini, but not in *Pantholops*.
4. *Aragoral mudejar* is from the site of La Roma 2, Spain, which is biostratigraphically correlated to European Neogene Mammal Zone MN 10, and, on the basis of high resolution local biostratigraphy and magnetostratigraphy, to an age of 8.9 Ma [99].

5. Belonging to either crown or stem Caprini, *Aragoral mudejar* provides a minimum age for the origin of the stem group. Given a lack of phylogenetic resolution surrounding much of the late Miocene record, the maximum limit is liberally extended back to allow for a much earlier origin of the stem Caprini. *Aragoral mudejar* could alternately have been used to provide a calibration for crown Caprini, using a normal probability distribution (as for crown Bovidae, crown Bovini, crown Tragelaphini above) with a range of 8.9 ± 2 Ma or thereabouts.

References

1. Gentry AW: **The subfamilies and tribes of the family Bovidae.** *Mamm Rev* 1992, **22:**1-32.
2. Geraads D: **Phylogenetic analysis of the tribe Bovini.** *Zool J Linn Soc* 1992, **104:**193-207.
3. Vrba ES: **New fossils of Alcelaphini and Caprinae (Bovidae; Mammalia) from Awash, Ethiopia, and phylogenetic analysis of Alcelaphini.** *Palaeontol Afr* 1997, **34:**127-198.
4. Vrba ES, Gatesy J: **New antelope fossils from Awash, Ethiopia, and phylogenetic analysis of Hippotragini (Bovidae, Mammalia).** *Palaeontol Afr* 1994, **31:**55-72.
5. Bibi F: **Evolution, Systematics, and Paleoecology of Bovinae (Mammalia: Artiodactyla) from the Late Miocene to the Recent.** *Ph.D. thesis.* Yale University, Geology & Geophysics; 2009.

6. Gibbard PL, Head MJ, Walker MJ: **Formal ratification of the Quaternary System/ Period and the Pleistocene Series/Epoch with a base at 2.58 Ma.** *J Quat Sci* 2010, **25:**96-102.

7. de Queiroz K: **Toward an integrated system of clade names.** *Syst Biol* 2007, **56:**956-974.

8. Nowak RM: *Walker's Mammals of the World, vol. 2.* 6 edn. Baltimore, USA: Johns Hopkins University Press; 1999.

9. Vrba ES, Schaller G (Eds.): **Antelopes, Deer, and Relatives**. New Haven: Yale University Press; 2000.

10. **IUCN red list of threatened species 2012.2 www.iucnredlist.org . Accessed April 2013.**

11. Heller R, Frandsen P, Lorenzen E, Siegismund H: **Are there really twice as many bovid species as we thought?** *Syst Biol* 2013, **doi: 10.1093/sysbio/syt004**.

12. Groves C, Grubb P: *Ungulate Taxonomy.* Baltimore: Johns Hopkins Univ.; 2011.

13. Parham JF, Donoghue PCJ, Bell CJ, Calway TD, Head JJ, Holroyd PA, Inoue JG, Irmis RB, Joyce WG, Ksepka DT: **Best practices for justifying fossil calibrations.** *Syst Biol* 2012, **61:**346-359.

14. Drummond AJ, Suchard MA, Xie D, Rambaut A: **Bayesian phylogenetics with BEAUti and the BEAST 1.7.** *Mol Biol Evol* 2012, **29:**1969-1973.

15. Müller J, Reisz RR: **Four well-constrained calibration points from the vertebrate fossil record for molecular clock estimates.** *Bioessays* 2005, **27:**1069-1075.

16. Gentry AW, Rössner GE, Heizmann EPJ: **Suborder Ruminantia.** In *The Miocene Land Mammals of Europe.* Edited by Rössner GE, Heissig K. Munich: Verlag Friedrich Pfeil; 1999: 225-258

17. Janis CM, Scott KM: **The interrelationships of higher ruminant families with special emphasis on the members of the Cervoidea.** *Am Mus Novit* 1987, **2893:**1-85.

18. Gilbert C, Ropiquet A, Hassanin A: **Mitochondrial and nuclear phylogenies of Cervidae (Mammalia, Ruminantia): systematics, morphology, and biogeography.** *Mol Phylogen Evol* 2006, **40:**101-117.

19. Hassanin A, Delsuc F, Ropiquet A, Hammer C, Jansen van Vuuren B, Matthee C, Ruiz-Garcia M, Catzeflis F, Areskoug V, Nguyen TT, Couloux A: **Pattern and timing of diversification of Cetartiodactyla (Mammalia, Laurasiatheria), as revealed by a comprehensive analysis of mitochondrial genomes.** *C R Biol* 2012, **335:**32-50.

20. Agustí J, Cabrera L, Garces M, Krijgsman W, Oms O, Pares JM: **A calibrated mammal scale for the Neogene of Western Europe. State of the art.** *Earth-Sci Rev* 2001, **52:**247-260.

21. Prothero DR: **Family Moschidae.** In *The Evolution of Artiodactyls.* Edited by Prothero D, Foss S. Baltimore: Johns Hopkins University Press; 2007: 221-226

22. Webb SD, Taylor BE: *The phylogeny of hornless ruminants and a description of the cranium of Archaeomeryx.* New York, NY, United States: American Museum of Natural History; 1980.

23. Janis CM, Scott KM: **The phylogeny of the Ruminantia (Artiodactyla, Mammalia).** In *The Phylogeny and Classification of Tetrapods; Volume 2, Mammals. Volume* 35b. Edited

by Benton MJ. London-New York, International: Academic Press [for the] Systematics Association; 1988: 273-282: *Systematics Association Special Volume*].

24. Su B, Wang Y-x, Lan H, Wang W, Zhang Y: **Phylogenetic study of complete cytochrome *b* genes in musk deer (Genus *Moschus*) using museum samples.** *Mol Phylogen Evol* 1999, **12:**241-249.

25. Hernández Fernández M, Vrba ES: **A complete estimate of the phylogenetic relationships in Ruminantia: a dated species-level supertree of the extant ruminants.** *Biol Rev Camb Philos Soc* 2005, **80:**269-302.

26. Hassanin A, Douzery EJP: **Molecular and morphological phylogenies of Ruminantia and the alternative position of the Moschidae.** *Syst Biol* 2003, **52:**206-228.

27. Gradstein FM, Ogg JG, Smith AG: *A Geologic Time Scale 2004.* Cambridge University Press; 2004.

28. Solounias N, Barry JC, Bernor RL, Lindsay EH, Raza SM: **The oldest bovid from the Siwaliks, Pakistan.** *J Vertebr Paleontol* 1995, **15:**806-814.

29. Solounias N, Moelleken SMC: **Dietary adaptations of two goat ancestors and evolutionary considerations.** *Géobios* 1992, **25:**797-809.

30. Azanza B, Morales J: ***Tethytragus* nov. gen et *Gentrytragus* nov. gen. Deux nouveaux Bovidés (Artiodactyla, Mammalia) du Miocène moyen.** *Proceedings, Koninklijke Nederlandse Akademie Van Wetenschappen* 1994, **B 97:**249-282.

31. Bibi F, Bukhsianidze M, Gentry AW, Geraads D, Kostopoulos DS, Vrba ES: **The fossil record and evolution of Bovidae: State of the field.** *Palaeontol Electron* 2009, **12:**10A: 11p.

32. Hassanin A, Douzery EJP: **The tribal radiation of the family Bovidae (Artiodactyla) and the evolution of the mitochondrial cytochrome b gene.** *Mol Phylogen Evol* 1999, **13:**227-243.

33. Gatesy J, Yelon D, Desalle R, Vrba ES: **Phylogeny of the Bovidae (Artiodactyla, Mammalia) Based on Mitochondrial Ribosomal DNA-Sequences.** *Mol Biol Evol* 1992, **9:**433-446.

34. Lowenstein JM: **Bovid relations based on serum immunology.** *S Afr J Sci* 1986, **82:**77-78.

35. Allard MW, Miyamoto MM, Jarecki L, Kraus F, Tennant MR: **DNA systematics and evolution of the artiodactyl family Bovidae.** *Proc Natl Acad Sci U S A* 1992, **89:**3972-3976.

36. Gatesy J, Amato G, Vrba ES, Schaller G, DeSalle R: **A cladistic analysis of mitochondrial ribosomal DNA from the Bovidae.** *Mol Phylogen Evol* 1997, **7:**303-319.

37. Kingdon J: *East African Mammals: An Atlas of Evolution in Africa: Volume III Part C (Bovids).* London: Academic Press; 1982.

38. Vrba ES, Schaller G: **Phylogeny of Bovidae based on behavior, glands, skulls, and postcrania.** In *Antelopes, Deer, and Relatives.* Edited by Vrba ES, Schaller G. New Haven: Yale University Press; 2000: 203-222

39. Ginsburg L, Morales J, Soria D: **Les Ruminantia (Artiodactyla, Mammalia) du Miocène des Bugti (Balouchistan, Pakistan).** *Estudios Geol* 2001, **57:**155-170.

40. Antoine P-O, Métais G, Orliac M, Crochet J-Y, Flynn LJ, Marivaux L, Rajpar AR, Roohi G, Welcomme J-L: **Mammalian Neogene biostratigraphy of the Sulaiman Province,**

Pakistan. In *Fossil Mammals of Asia: Neogene Biostratigraphy and Chronology.* Edited by Wang X, Flynn LJ, Fortelius M. New York: Columbia University Press; 2013: 400-422

41. Johnson NM, Stix J, Tauxe L, Cerveny PF, Tahirkheli RAK: **Paleomagnetic chronology, fluvial processes, and tectonic implications of the Siwalik deposits near Chinji Village, Pakistan.** *The Journal of Geology* 1985, **93:**27-40.

42. Benton MJ, Donoghue PCJ: **Paleontological evidence to date the tree of life.** *Mol Biol Evol* 2007, **24:**26.

43. Bibi F: **Origin, paleoecology, and paleobiogeography of early Bovini.** *Palaeogeogr Palaeoclimatol Palaeoecol* 2007, **248:**60-72.

44. Barry JC, Morgan ME, Flynn LJ, Pilbeam D, Behrensmeyer AK, Raza SM, Khan IA, Badgley C, Hicks J, Kelley J: **Faunal and environmental change in the late Miocene Siwaliks of northern Pakistan.** *Paleobiology* 2002, **28(S2):**1-71.

45. Badgley C, Barry JC, Morgan ME, Nelson SV, Behrensmeyer AK, Cerling TE, Pilbeam D: **Ecological changes in Miocene mammalian record show impact of prolonged climatic forcing.** *Proceedings of the National Academy of Sciences* 2008, **105:**12145-12149.

46. Pilgrim GE: **The fossil Bovidae of India.** *Palaeontol Indica* 1939, **NS 26:**1-356.

47. Dung VV, Giao PM, Chinh NN, Tuoc D, Arctander P, Mackinnon J: **A new species of living bovid from Vietnam.** *Nature* 1993, **363:**443-445.

48. Hassanin A, Ropiquet A: **Molecular phylogeny of the tribe Bovini (Bovidae, Bovinae) and the taxonomic status of the Kouprey, *Bos sauveli* Urbain 1937.** *Mol Phylogen Evol* 2004, **33:**896-907.

49. Hassanin A, Douzery EJP: **Evolutionary affinities of the enigmatic saola (*Pseudoryx nghetinhensis*) in the context of the molecular phylogeny of Bovidae.** *Proceedings of the Royal Society of London Series B-Biological Sciences* 1999, **266:**893-900.

50. Thomas H: **Anatomie crânienne et relations phylogénétiques du nouveau bovidé (*Pseudoryx nghetinensis*) découvert dans la cordillère annamitique au Vietnam.** *Mammalia* 1994, **58:**453-481.

51. Gatesy J, Arctander P: **Hidden morphological support for the phylogenetic placement of *Pseudoryx nghetinhensis* with bovine bovids: A combined analysis of gross anatomical evidence and DNA sequences from five genes.** *Syst Biol* 2000, **49:**515-538.

52. Hassanin A, An J, Ropiquet A, Nguyen TT, Couloux A: **Combining multiple autosomal introns for studying shallow phylogeny and taxonomy of Laurasiatherian mammals: Application to the tribe Bovini (Cetartiodactyla, Bovidae).** *Mol Phylogen Evol* 2012.

53. Thomas H: **Les Bovidés anté-hipparions des Siwaliks inférieurs (Plateau du Potwar, Pakistan).** *Mémoires de la Société Géologique de France, Nouvelle Série* 1984, **145:**1-68.

54. Akhtar M: ***Pachyportax giganteus*, new species (Mammalia: Artiodactyla: Bovidae) from the Dhok Pathan, District Chakwal, Punjab, Pakistan.** *Pak J Zool* 1995, **27:**337-340.

55. Haile-Selassie Y, Vrba ES, Bibi F: **Bovidae.** In *Ardipithecus kadabba: Late Miocene Evidence from the Middle Awash, Ethiopia.* Edited by Haile-Selassie Y, WoldeGabriel G. Berkeley: University of California Press; 2009: 277-330

56. Thomas H: **Les bovidés du Miocène supérieur des couches de Mpesida et de la formation de Lukeino (district de Baringo, Kenya).** In *Proceedings of the 8th Panafrican Congress of Prehistory and Quaternary Studies (Nairobi 1977).* Edited by Leakey REF, Ogot BA; 1980: 82-91

57. Kostopoulos DS, Koufos GD: ***Pheraios chryssomallos*, gen. et sp nov (Mammalia, Bovidae, Tragelaphini), from the late Miocene of Thessaly (Greece): Implications for tragelaphin biogeography.** *J Vertebr Paleontol* 2006, **26:**436-445.

58. Gentry AW: **Bovidae.** In *Cenozoic Mammals of Africa.* Edited by Werdelin L, Sanders WJ. Berkeley: University of California Press; 2010: 747-803

59. Willows-Munro S, Robinson TJ, Matthee CA: **Utility of nuclear DNA intron markers at lower taxonomic levels: Phylogenetic resolution among nine *Tragelaphus* spp.** *Mol Phylogen Evol* 2005, **35:**624-636.

60. Deino AL, Tauxe L, Monaghan M, Hill A: **Ar-40/Ar-30 geochronology and paleomagnetic stratigraphy of the Lukeino and lower Chemeron Formations at Tabarin and Kapcheberek, Tugen Hills, Kenya.** *J Hum Evol* 2002, **42:**117-140.

61. WoldeGabriel G, Haile-Selassie Y, Renne PR, Hart WK, Ambrose SH, Asfaw B, Heiken G, White T: **Geology and palaeontology of the Late Miocene Middle Awash valley, Afar rift, Ethiopia.** *Nature* 2001, **412:**175-178.

62. Harris JM: **Bovidae from the Lothagam succession.** In *Lothagam: The Dawn of Humanity in Eastern Africa.* Edited by Leakey MG, Harris JM. New York: Columbia University Press; 2003: 531-579

63. Bibi F: ***Tragelaphus nakuae*: Evolutionary change, biochronology, and turnover in the African Plio-Pleistocene.** *Zool J Linn Soc* 2011, **162:**699-711.

64. Arambourg C: **Antilopes nouvelles du pleistocene ancien de l'Omo (Abyssinie).** *Mus Natl Hist nat, Paris, B s 2* 1941, **13:**339-347.

65. Gentry AW: **The Bovidae of the Omo Group deposits, Ethiopia (French and American collections).** In *Les faunes Plio-Pléistocènes de la basse Vallée de l'Omo (Ethiopie); I: Perissodactyles-Artiodactyles (Bovidae). Volume* 1985. Edited by Coppens Y, Howell FC. Paris, France: CNRS; 1985: 119-191: *Cahiers de Paléontologie.*].

66. Harris JM: **Family Bovidae.** In *Koobi Fora Research Project Volume III. Volume* 3. Edited by Harris JM. Oxford: Clarendon Press; 1991: 139-320.[Leakey REF (Series Editor): *Koobi Fora Research Project*].

67. Geraads D, Melillo S, Haile-Selassie Y: **Middle Pliocene Bovidae from hominid-bearing sites in the Woranso-Mille area, Afar region, Ethiopia.** *Palaeontol Afr* 2009, **44:**59-70.

68. WoldeGabriel G, Endale T, White TD, Thouveny N, Hart WK, Renne PR, Asfaw B: **The Role of Tephra Studies in African Paleoanthropology as Exemplified by the Sidi Hakoma Tuff.** *J Afr Earth Sci* 2013, **77:**41-58.

69. Reed K, Bibi F: **Fossil Tragelaphini (Artiodactyla: Bovidae) from the Hadar Formation, Afar Regional State, Ethiopia.** *J Mamm Evol* 2011, **1:**57-69.

70. Vrba ES: **A possible ancestor of the living waterbuck and lechwes: *Kobus basilcookei* sp. nov. (Reduncini, Bovidae, Artiodactyla) from the Early Pliocene of the Middle Awash, Ethiopia.** *Trans R Soc S Afr* 2006, **61:**63-74.

71. Vrba ES, Vaisnys JR, Gatesy JE, Desalle R, Wei KY: **Analysis of pedomorphosis using allometric characters – the example of Reduncini antelopes (Bovidae, Mammalia).** *Syst Biol* 1994, **43:**92-116.

72. Birungi J, Arctander P: **Molecular systematics and phylogeny of the Reduncini (Artiodactyla: Bovidae) inferred from the analysis of mitochondrial cytochrome *b* gene sequences.** *J Mamm Evol* 2001, **8:**125-147.

73. Renne PR, WoldeGabriel G, Hart WK, Heiken G, White TD: **Chronostratigraphy of the Miocene-Pliocene Sagantole Formation, Middle Awash Valley, Afar rift, Ethiopia.** *Geol Soc Am Bull* 1999, **111:**869-885.

74. Feibel CS, Brown FH, Mcdougall I: **Stratigraphic Context of Fossil Hominids from the Omo Group Deposits - Northern Turkana Basin, Kenya and Ethiopia.** *Am J Phys Anthropol* 1989, **78:**595-622.

75. Geraads D, Blondel C, Likius A, Mackaye HT, Vignaud P, Brunet M: **New Hippotragini (Bovidae, Mammalia) from the late Miocene of Toros-Menalla (Chad).** *J Vertebr Paleontol* 2008, **28:**231-242.

76. Vignaud P, Duringer P, Mackaye HT, Likius A, Blondel C, Boisserie JR, de Bonis L, Eisenmann V, Etienne ME, Geraads D, et al: **Geology and palaeontology of the Upper Miocene Toros-Menalla hominid locality, Chad.** *Nature* 2002, **418:**152-155.

77. Lebatard AE, Bourles DL, Duringer P, Jolivet M, Brauchert R, Carcaillet J, Schuster M, Arnaud N, Monie P, Lihoreau F, et al: **Cosmogenic nuclide dating of *Sahelanthropus tchadensis* and *Australopithecus bahrelghazali*: Mio-Pliocene hominids from Chad.** *Proceedings of the National Academy of Sciences* 2008, **105:**3226-3231.

78. Gentry AW: **The earliest goats and other antelopes from the Samos *Hipparion* fauna.** *Bulletin of the British Museum (Natural History) Geology Series* 1971, **20:**229-296.

79. Gentry AW: **Caprinae and Hippotragini (Bovidae, Mammalia) in the Upper Miocene.** In *Antelopes, Deer, and Relatives.* Edited by Vrba ES, Schaller G. New Haven: Yale University Press; 2000: 65-83

80. Bosscha Erdbrink DP: ***Protoryx* from three localities east of Maragheh, N.W. Iran.** *Proceedings Koninklijke Nederlandse Akademie Van Wetenschappen Ser B* 1988, **91:**101-159.

81. Gentry AW: **Bovidae.** In *Paleontology and Geology of Laetoli: Human Evolution in Context Volume 2.* Edited by Harrison T. New York: Springer; 2011: 363-465

82. Deino AL: **40Ar/39Ar dating of Laetoli, Tanzania.** In *Paleontology and Geology of Laetoli: Human Evolution in Context.* Edited by Harrison T. Dordrecht: Springer; 2011: 77-97

83. Gentry AW: **Fossil Bovidae (Mammalia) from Langebaanweg, South Africa.** *Ann S Afr Mus* 1980, **79:**213-337.

84. Faith JT, Choiniere JN, Tryon CA, Peppe DJ, Fox DL: **Taxonomic status and paleoecology of *Rusingoryx atopocranion* (Mammalia, Artiodactyla), an extinct Pleistocene bovid from Rusinga Island, Kenya.** *Quatern Res* 2011, **75:**697-707.

85. Hendey QB: **Geological succession at Langebaanweg, Cape Province, and global events of the late Tertiary.** *S Afr J Sci* 1981, **77:**33-38.

86. Arctander P, Johansen C, Coutellec-Vreto MA: **Phylogeography of three closely related African bovids (tribe Alcelaphini).** *Mol Biol Evol* 1999, **16:**1724-1739.

87. Flagstad ÿ, Syversten PO, Stenseth NC, Jakobsen KS: **Environmental change and rates of evolution: the phylogeographic pattern within the hartebeest complex as related to climatic variation.** *Proc R Soc Lond B Biol Sci* 2001, **268:**667.

88. Clark JD, de Heinzelin J, Schick KD, Hart WK, White TD, WoldeGabriel G, Walter RC, Suwa G, Asfaw B, Vrba E: **African *Homo erectus*: old radiometric ages and young Oldowan assemblages in the Middle Awash Valley, Ethiopia.** *Science* 1994, **264:**1907-1910.

89. Gentry AW, Gentry A: **Fossil Bovidae (Mammalia) of Olduvai Gorge, Tanzania; Part I.** *Bulletin of the British Museum Natural History Geology Series* 1978, **29:**p. 289-446.

90. Gentry AW: **The Semliki Fossil Bovids.** In *Evolution of Environments and Hominidae in the African Western Rift Valley.* Edited by Boaz NT. Martinsville: Virginia Museum of Natural History; 1990: 225-234: *Virginia Museum of Natural History Memoir*].

91. Harris JM, Brown FH, Leakey MG: **Stratigraphy and paleontology of Pliocene and Pleistocene localities west of Lake Turkana, Kenya.** *Contributions in Science* 1988, **399:**1-128.

92. Deino AL: **40Ar/39Ar dating of Bed I, Olduvai Gorge, Tanzania, and the chronology of early Pleistocene climate change.** *J Hum Evol* 2012, **63:**251-273.

93. Hay RL: *Geology of the Olduvai Gorge: A Study of Sedimentation in a Semiarid Basin.* Berkeley: University of California Press; 1976.

94. Alcala L, Morales J, Moyà-Solà S: **El registro fosil neogeno de los bovidos (Artiodactila, Mammalia) de Espana.** *Paleontol Evolucio* 1990, **23:**67-73.

95. Ropiquet A, Hassanin A: **Molecular phylogeny of caprines (Bovidae, Antilopinae): the question of their origin and diversification during the Miocene.** *J Zool Syst Evol Res* 2005, **43:**49-60.

96. Ropiquet A, Hassanin A: **Molecular evidence for the polyphyly of the genus Hemitragus (Mammalia, Bovidae).** *Mol Phylogen Evol* 2005, **36:**154-168.

97. Ropiquet A, Hassanin A: **Hybrid origin of the Pliocene ancestor of wild goats.** *Mol Phylogen Evol* 2006, **41:**395-404.

98. Hassanin A, Ropiquet A, Couloux A, Cruaud C: **Evolution of the mitochondrial genome in mammals living at high altitude: new insights from a study of the tribe Caprini (Bovidae, Antilopinae).** *J Mol Evol* 2009, **68:**293-310.

99. Van Dam JA, Alcalá L, Zarza AA, Calvo JP, Garcés M, Krijgsman W: **The upper Miocene mammal record from the Teruel-Alfambra region (Spain). The MN system and continental stage/age concepts discussed.** *J Vertebr Paleontol* 2001, **21:**367-385.
